# Adipocyte-derived FABP4 promotes metabolism-associated steatotic liver–induced hepatocellular carcinoma by driving ITGB1-mediated **β**-catenin activation

**DOI:** 10.1172/JCI182322

**Published:** 2025-12-15

**Authors:** Carmen Oi Ning Leung, Shilpa Gurung, Katherine Po Sin Chung, Rainbow Wing Hei Leung, Martina Mang Leng Lei, Mandy Sze Man Chan, Gregory Kenneth Muliawan, Shakeel Ahmad Khan, Xue Qian Wu, Jun Yu, Hui Lian Zhu, Yin Ying Lu, Stephanie Ma, Xiaoping Wu, Ruby Lai Chong Hoo, Terence Kin Wah Lee

**Affiliations:** 1 Department of Applied Biology and Chemical Technology, The Hong Kong Polytechnic University, Hong Kong, China.; 2 Institute of Digestive Disease and Department of Medicine and Therapeutics, State Key Laboratory of Digestive Disease, Li Ka Shing Institute of Health Sciences, The Chinese University of Hong Kong, Hong Kong, China.; 3 School of Public Health, Sun Yat Sen University, Guangzhou, China.; 4 Comprehensive Liver Cancer Center, The Fifth Medical Center of PLA General Hospital, Beijing, China.; 5 School of Biomedical Sciences, Li Ka Shing Faculty of Medicine,; 6 State Key Laboratory of Liver Research, and; 7 Department of Pharmacology and Pharmacy, Li Ka Shing Faculty of Medicine, The University of Hong Kong, Hong Kong, China.; 8 Research Institute for Future Food, The Hong Kong Polytechnic University, Hong Kong, China.

**Keywords:** Hepatology, Oncology, Adipose tissue, Integrins, Liver cancer

## Abstract

Metabolic dysfunction–associated steatotic liver disease–induced (MASLD-induced) hepatocellular carcinoma (HCC) is an emerging malignancy linked to excessive accumulation of adipose tissue and hepatic fat. Understanding the role of adipocytes in the development of MASLD-induced HCC is crucial. In an in vitro coculture system, differentiated adipocytes were found to enhance cancer stemness and drug resistance in HCC through paracrine signaling. Fatty acid–binding protein 4 (FABP4) was preferentially secreted by adipocytes, and recombinant FABP4 further augmented the cancer stem cell (CSC) properties of HCC cells. Notably, *Fabp4^–/–^* mice exhibited a marked delay in the progression of MASLD-HCC, which correlated with the increased HCC risk observed in MASLD patients with elevated FABP4 expression. Mass spectrometry analysis identified integrin β 1 (ITGB1) as a binding partner of FABP4. These data, together with a substantial downregulation of the Wnt/β-catenin pathway in *Fabp4^–/–^* mouse tumors, revealed that FABP4 augmented liver CSC functions by activating PI3K/AKT/β-catenin signaling via ITGB1. We developed an anti-FABP4 neutralizing antibody that successfully inhibited FABP4-driven CSC functions and suppressed MASLD-induced HCC. In conclusion, our findings indicate that adipocyte-derived FABP4 plays a critical role in the development of MASLD-induced HCC and targeting the ITGB1/PI3K/AKT/β-catenin signaling cascade may offer a promising approach to combat this aggressive disease.

## Abstract 2

No approved drugs exist for MASLD-induced HCC. Targeting adipocyte-derived FABP4 may offer a new treatment approach, blocking cancer stem cell-driven tumor growth.

## Graphical abstract

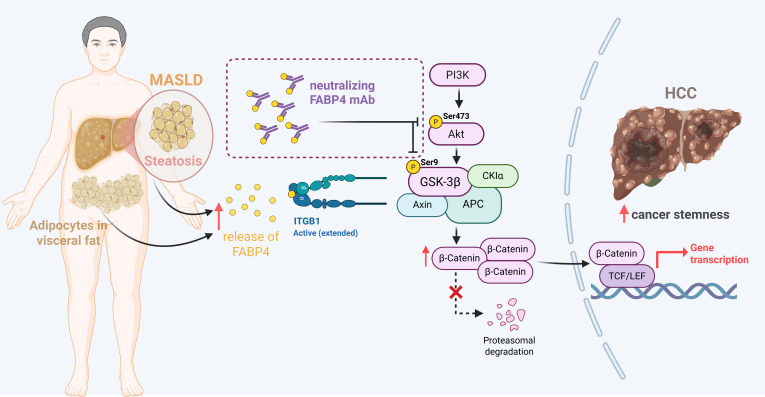

## Introduction

Liver cancer ranks as the third deadliest malignancy worldwide (1). Hepatocellular carcinoma (HCC), which accounts for approximately 90% of primary liver cancers, typically arises in a background of chronic liver disease caused by HBV or HCV infection, alcohol-associated liver diseases, or the increasingly common metabolic dysfunction–associated steatotic liver disease (MASLD) (2, 3). The global prevalence of MASLD is approximately 25%, and MASLD has become not only the most common chronic liver disease but also a public health crisis (4). It is diagnosed as MASLD if hepatic steatosis is accompanied by either obesity or overweight, type 2 diabetes mellitus, or evidence of metabolic dysregulation (5, 6). Metabolic dysfunction–associated steatohepatitis (MASH), one of the large subtypes in MASLD (7), is diagnosed when there is inflammatory activity and hepatocyte injury present in a fatty liver tissue (2, 8, 9). With the increasing incidence of obesity and diabetes, MASH is becoming the fastest growing etiology of HCC (10–12). Although recent studies suggested that the prevalence of HCC increased 11.5-fold in patients with MASH (13), the pathogenesis and molecular mechanisms underlying the onset of these conditions are still poorly understood. Currently, therapeutic strategies specifically targeting MASLD-induced HCC are limited (14, 15). Standard treatments for virus-associated HCC, such as molecular targeted therapies, antiangiogenic therapies, and immune checkpoint inhibitors, are also employed for MASLD-induced HCC but are frequently ineffective (16). Therefore, identifying new therapeutic targets and effective strategies for MASLD-induced HCC is crucial.

While MASLD is recognized as a risk factor for HCC, our understanding of its impact on the development of HCC is still limited. Current studies suggest that MASLD induces combined stresses, such as oxidative stress and chronic inflammation, promoting malignant transformation of hepatocytes through regenerative proliferation and fibrotic remodeling, both of which contribute to tumor development (17–20). The progressive accumulation of cellular defects, including mutations, and changes in the microenvironment, such as inflammation and fibrosis, ultimately leads to malignant transformation and tumor emergence. Thus, the risk of HCC increases as the disease progresses from early MASLD to MASH. Nevertheless, MASLD is a condition characterized by liver fat accumulation in individuals with diabetes, obesity, high blood pressure, or high cholesterol who consume little to no alcohol (21). This phenomenon suggested that visceral adipose tissue and hepatic fat might contribute to the development of MASLD-induced HCC. Adipose tissue regulates energy balance by managing excess caloric intake associated with obesity (22). Its adaptation through excessive triglyceride storage leads to metabolic disturbances mediated by factors such as abnormal adipokine secretion, contributing to MASLD (23). Interestingly, we and others have also demonstrated a link between mouse liver cancer stem cells (CSCs) and obesity-mediated steatohepatitis (24, 25). Nonetheless, the link between HCC stemness and the interplay of adipocytes in MASLD is unclear.

Here, we utilized adipocytes differentiated from human visceral preadipocytes and revealed that the adipocyte secretome was critically involved in promoting the function of liver CSCs, while the CSC-enhancing effect was further enhanced upon coculturing with HCC cells. Orbitrap analysis revealed that fatty acid–binding protein 4 (FABP4) played a crucial role in this mechanism. Functional studies showed that secreted FABP4 augmented HCC tumor growth by modulating the PI3K/AKT/β-catenin signaling pathway through its interaction with ITGB1. These data are consistent with the delayed development of MASLD-HCC in *Fabp4^–/–^* mice. We developed a monoclonal neutralizing antibody that specifically targets FABP4, which not only efficiently blocked FABP4-driven CSC functions, but also efficiently suppressed tumor formation in a MASLD-HCC mouse model.

## Results

### Adipocytes, not preadipocytes, enhance liver cancer stemness and drug resistance in HCC cells.

**Figure 1:**
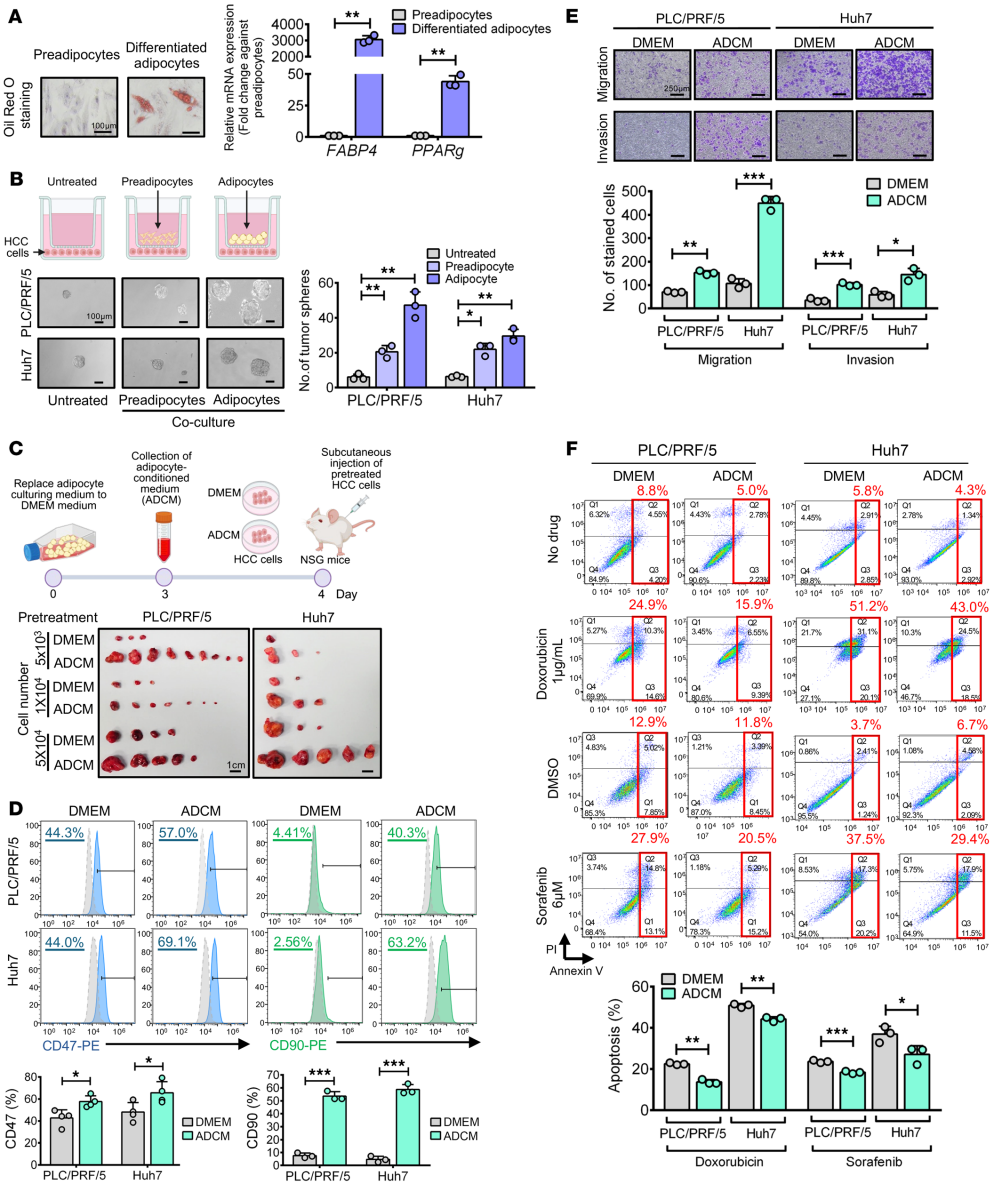
Adipocytes enhanced self-renewal and tumorigenicity and drove drug resistance in HCC cells in a paracrine manner. (**A**) Representative images of intracellular lipids in adipocytes after differentiation. mRNA expression of functional genes of adipocytes (*n* = 3). Scale bars: 100 μm. (**B**) Schematic diagram of coculturing setup for sphere formation. Representative images of sphere formation (*n* = 3). Scale bars: 100 μm. (**C**) Schematic diagram showing the workflow of adipocyte-condition medium (ADCM) collection, pretreatment of HCC cells, and subcutaneous injection of cells into NOD/SCID gamma (NSG) mice. Representative images of tumors. Scale bars: 1 cm. (**D**) Expression of liver CSC markers (*n* = 3-4). (**E**) The migration and invasive abilities of HCC cells were evaluated (*n* = 3). Representative images of stained cells. Scale bar: 250 μm. (**F**) Apoptosis of pretreated (DMEM or ADCM) HCC cells induced by doxorubicin or sorafenib (*n* = 3). Data represent mean ± SD. *P < 0.05, **P < 0.01, ***P < 0.001, 2-tailed *t* test.

An increase in visceral fat results in increased production of proinflammatory adipokines (26), and this dysregulation of adipokines via visceral adipose tissue contributes to the development of MASH (27); hence, we established human adipocytes from in vitro differentiation of commercially available human visceral preadipocyte cells. Successful establishment of functional visceral adipocytes was evidenced by the presence of intracellular lipids and upregulation of specific genes related to adipocyte differentiation and adipogenesis, including *FABP4* and *PPARG,* compared with their preadipocyte counterparts (Figure 1A). Coculturing these adipocytes with HCC cells in a Transwell experimental setup promoted significant increase in self-renewal ability of HCC cells in comparison to coculture in untreated condition or treated with undifferentiated preadipocytes (Figure 1B). This result suggested that adipocytes regulate liver CSCs via paracrine secretion. To verify this, we collected the conditioned medium of adipocytes (ADCM) for an in vivo limiting dilution assay. Pretreatment of HCC cells with ADCM increased the size and number of HCC tumors (Figure 1C and Supplemental Table 1; supplemental material available online with this article; https://doi.org/10.1172/JCI182322DS1). In addition, pretreatment of HCC cells with ADCM also expression of liver CSC markers, including CD47 and CD90 (Figure 1D); migration and invasion (Figure 1E); and resistance to doxorubicin and sorafenib (Figure 1F). All these results suggest that adipocyte secretomes exert CSC-enhancing effects in a paracrine manner.

### Mass spectrometry profiling revealed that FABP4 is preferentially secreted by adipocytes in coculture with HCC cells.

**Figure 2:**
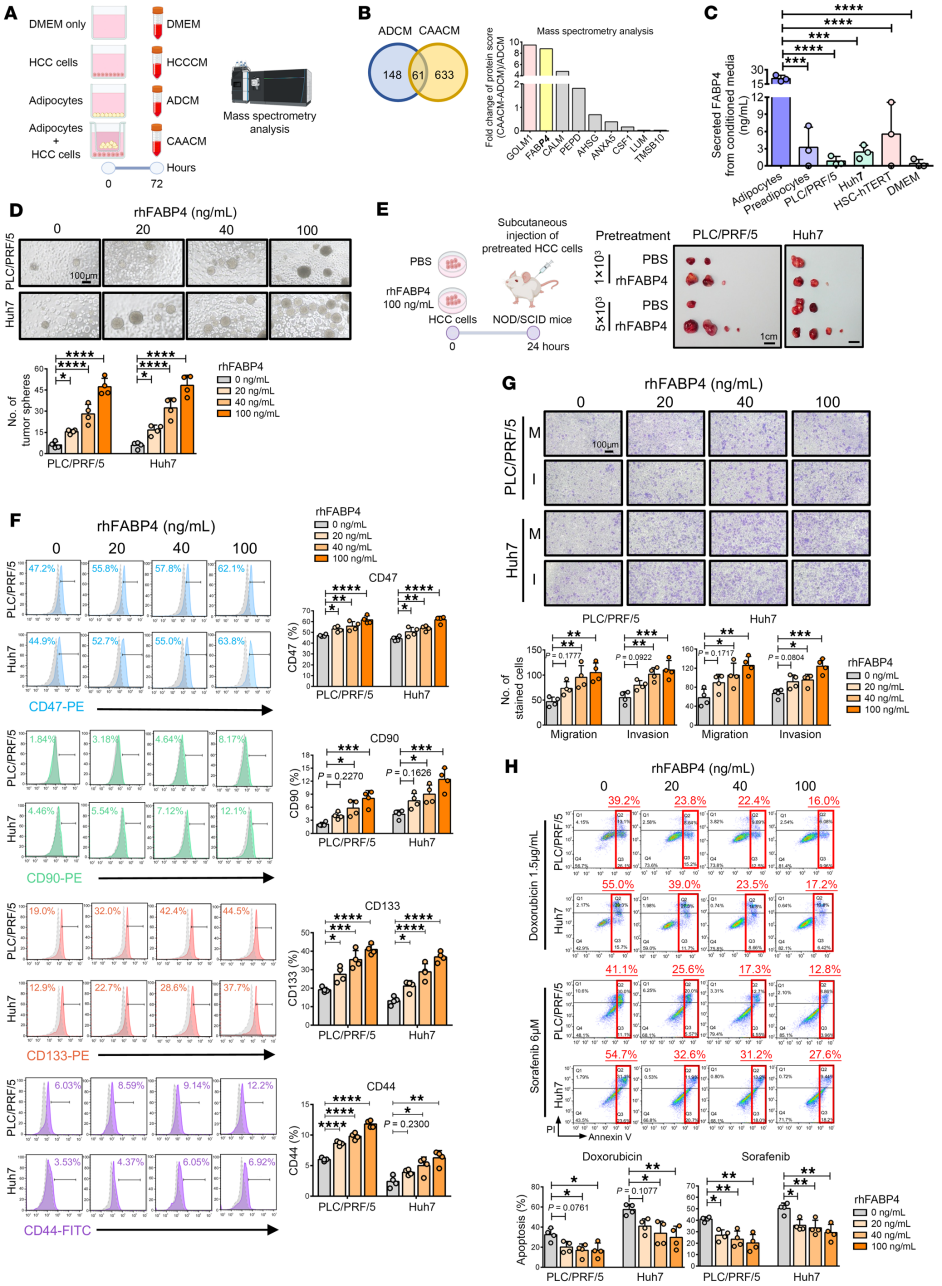
Identification of FABP4 as an important secretome conferring CSC properties in HCC cells. (**A**) Schematic diagram illustrating the workflow for collection of adipocyte-conditioned medium (ADCM), HCC cells only (HCCCM), ADCM cotreated with HCC cells (CAACM), and DMEM only (control) for mass spectrometry analysis. For further details, see Supplemental Figure 1A. (**B**) Venn diagram illustrating 61 proteins commonly found in both ADCM and CAACM (*n* = 1). Nine targets were found to be further upregulated in CAACM. (**C**) Secreted FABP4 in 72 hour-CM (*n* = 3). (**D**) Sphere formation assay demonstrated the role of recombinant human FABP4 (rhFABP4) in regulation of self-renewal ability of HCC cells (*n* = 4). Representative images of tumorspheres. Scale bar: 100 μm. (**E**) Schematic diagram of pretreatment of HCC cells with rhFABP4 for subcutaneous inoculation using NOD/SCID mice. Representative photos of tumors. Scale bars: 1 cm. (**F**) Expression of liver CSC markers including CD44, CD47, CD90, and CD133 (*n* = 4). (**G**) Migration (M) and invasive (I) abilities of HCC cells were evaluated (*n* = 4). Representative images of stained cells. Scale bar: 100 μm. (**H**) Apoptosis of pretreated HCC cells with either PBS (rhFABP4 0 ng/mL) or rhFABP4 induced by doxorubicin or sorafenib was measured (*n* = 4). Data represent mean ± SD. *P < 0.05, **P < 0.01, ***P < 0.001, ****P < 0.0001. **C**, **D**, and **F**–**H**: 1-way ANOVA followed by Tukey’s multiple-comparison test.

In our pilot study, we found that adipocyte secretomes promoted self-renewal of HCC cells, and the CSC-enhancing effect was further enhanced upon incubation with conditioned medium from coculturing of ADCM and HCC cells (stimulated adipocyte CM, CAACM) (Supplemental Figure 1A). Based on these findings, we aimed to determine the secretory factors crucial for driving CSC functions by identifying those that are not only released by adipocytes but also, and most importantly, further enhanced in coculture with HCC cells. For this purpose, we collected ADCM and CAACM and profiled them by Orbitrap mass spectrometry analysis (Figure 2A). Using DMEM as a control, we identified the top 22 adipocyte-specific secretory proteins from ADCM (Supplemental Table 2). Among these candidates, 9 were found to be further upregulated in CAACM (Figure 2B), making them our potential targets of interest. Among the 2 targets (GOLM1 and FABP4) with the highest fold increase, we selected FABP4 for further functional characterization, as GOLM1 was found to be abundantly secreted in CM of HCC cells (HCCCM) based on our mass spectrometry data (Supplemental Table 2). Next, we compared secretory FABP4 levels in human visceral preadipocytes, differentiated adipocytes, HCC cell lines, and activated human hepatic stellate cells (hTERT-HSCs). Both HCC cell lines and hTERT-HSCs produced negligible or no FABP4, similar to negative control DMEM-treated cells and preadipocytes, whereas differentiated adipocytes produced a marked level of FABP4, indicating adipocytes as the primary FABP4 source (Figure 2C). Furthermore, we observed about a 4-fold increase in the secretory level of FABP4 (a mean of 95 ng/mL) in CAACM compared with that in ADCM based on the conditioned media collection shown in Supplemental Figure 1, B and C. To investigate the functional role of adipocyte-derived FABP4 in the regulation of liver CSC properties, we examined the CSC properties of HCC cells by administering recombinant human FABP4 protein (rhFABP4) at 20 ng/mL, 40 ng/mL, and 100 ng/mL, where these concentrations represent physiological levels from ADCM and CAACM. rhFABP4 promoted liver CSC properties, including self-renewal (Figure 2D), tumorigenicity (Figure 2E and Supplemental Table 3), expression of liver CSC markers (Figure 2F), migration and invasion (Figure 2G), and resistance to doxorubicin and sorafenib treatment (Figure 2H). These data, together with our functional observation showing attenuation of the effects of ADCM on liver CSC properties in HCC upon treatment with the FABP4-specific inhibitor BMS-309403 (Supplemental Figure 2, A–D), further suggested the critical role of adipocyte-derived FABP4 in the promotion of cancer stemness.

### Genetic ablation of FABP4 delays the onset of tumor development in a MASLD-HCC mouse model.

**Figure 3:**
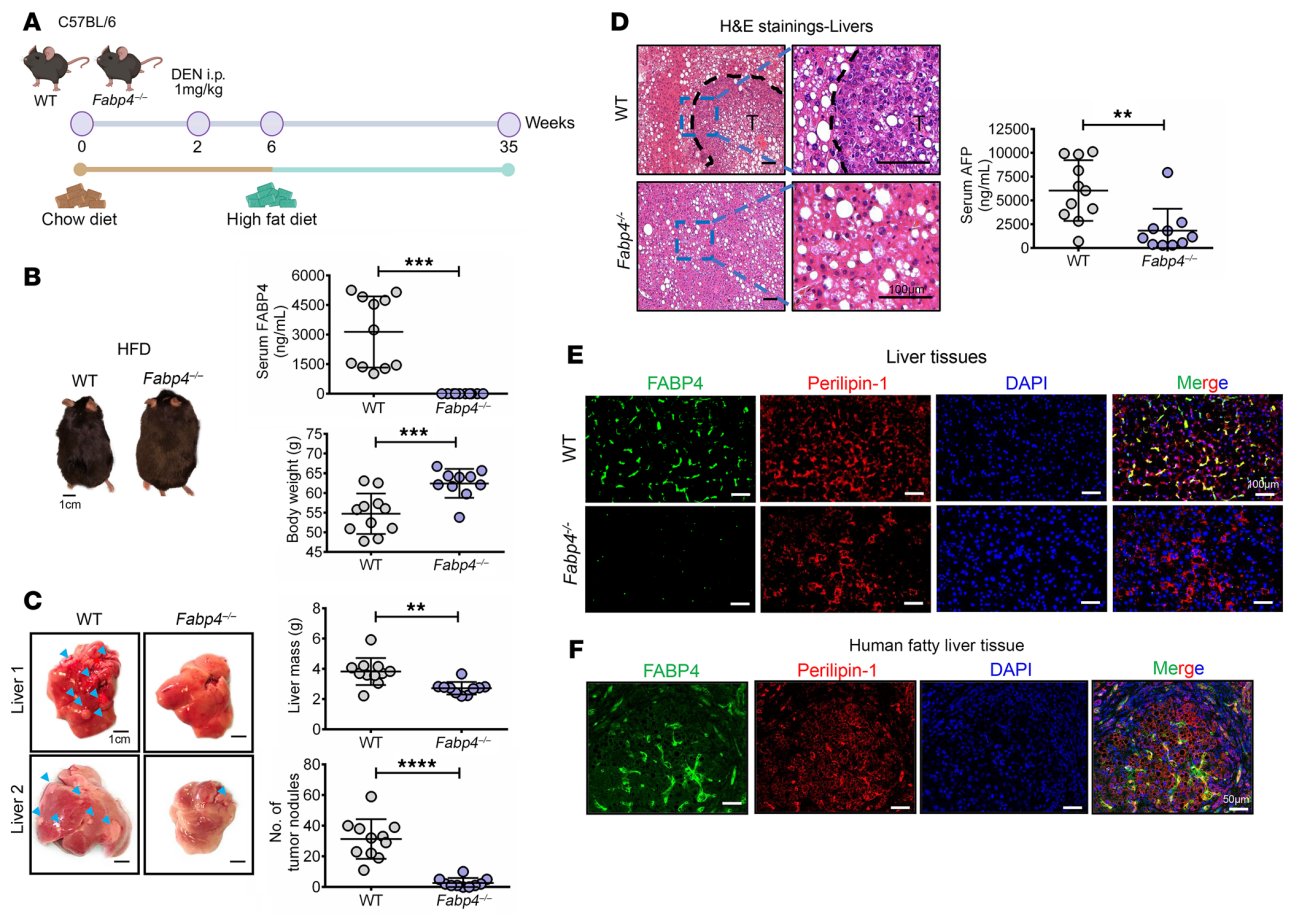
FABP4 deficiency delayed the onset of tumor development in DEN-injected and HFD-induced MASLD-HCC mouse model. (**A**) Schematic diagram showing establishment of DEN-injected and HFD-induced MASLD-HCC mouse model. (**B**) Representative photos of WT and *Fapb4^–/–^* mice after feeding with HFD for 29 weeks. Scale bar: 1 cm. Expression of FABP4 in mouse sera and body weight (WT *n* = 11 mice and *Fabp4^–/–^* *n* = 10 mice). (**C**) Representative photos of livers. Graphs showing liver mass and number of tumor nodules. Blue arrowheads: tumor nodules. Scale bars: 1 cm. (**D**) Representative images of H&E staining of the tumors (T). Expression of AFP in mouse sera. Scale bars: 100 μm. (**E** and **F**) Expression of FABP4 (green) and perilipin-1 (red) in mouse livers and human fatty liver tissue. DAPI: nuclei, blue; Scale bar: 100 μm and 50 μm, respectively. Data represent mean ± SD. **P < 0.01, ***P < 0.001, ****P < 0.0001, 2-tailed *t* test.

To determine the role of FABP4 in MASLD-HCC, we subjected *Fabp4*-KO (*Fabp4^–/–^*) male mice and their WT littermates to *N*-nitrosodiethylamine (DEN) treatment at 2 weeks of age and fed them a high-fat diet (HFD) at 6 weeks of age for 29 weeks to induce MASLD-HCC formation (Figure 3A). We did not detect any secreted FABP4 in the *Fabp4^–/–^* mice, and these mice exhibited significantly greater body weight than their WT counterparts (Figure 3B). Consistent with our hypothesis, we observed impaired tumor growth in *Fabp4^–/–^* mice, as evidenced by a significant decrease in liver mass and the number of tumor nodules (Figure 3C). The suppression of tumor growth was also reflected by histological staining and a significant decrease in the serum α-fetoprotein (AFP) level (Figure 3D). Interestingly, genetic loss of FABP4 attenuated liver steatosis, inflammation, and fibrosis, as demonstrated by steatosis and lobular inflammation scores, lipid deposition, reduced expression of inflammatory and fibrotic genes, collagen deposition, α-SMA expression, F4/80 macrophages, and M1/M2 macrophage ratio (Supplemental Figure 3, A–H). Our qPCR analysis revealed visceral adipocytes as the primary distant source of secreted FABP4 in mice with MASLD, contributing to the above liver phenotypes (Supplemental Figure 3I). This aligns with protein atlas single-cell data showing that adipocytes had the highest *FABP4* expression among the cell types examined (Supplemental Figure 3J). Furthermore, we observed preferential colocalization of FABP4 with perilipin-1^+^ (PLIN1^+^) compared with CD31^+^ endothelial cells and F4/80^+^ macrophages, indicating that FABP4 was preferentially expressed in lipid droplets within steatotic hepatocytes (Figure 3E and Supplemental Figure 3K). The specificity of FABP4 as found in the lipid droplets was also evident in human fatty liver tissue (Figure 3F). These findings indicate that visceral adipocytes and lipid droplet-rich in steatotic hepatocytes were the major sources of secretory FABP4 in MASLD-induced mice.

### FABP4 is sporadically expressed in MASLD-induced HCC patients with clinical significance.

**Figure 4:**
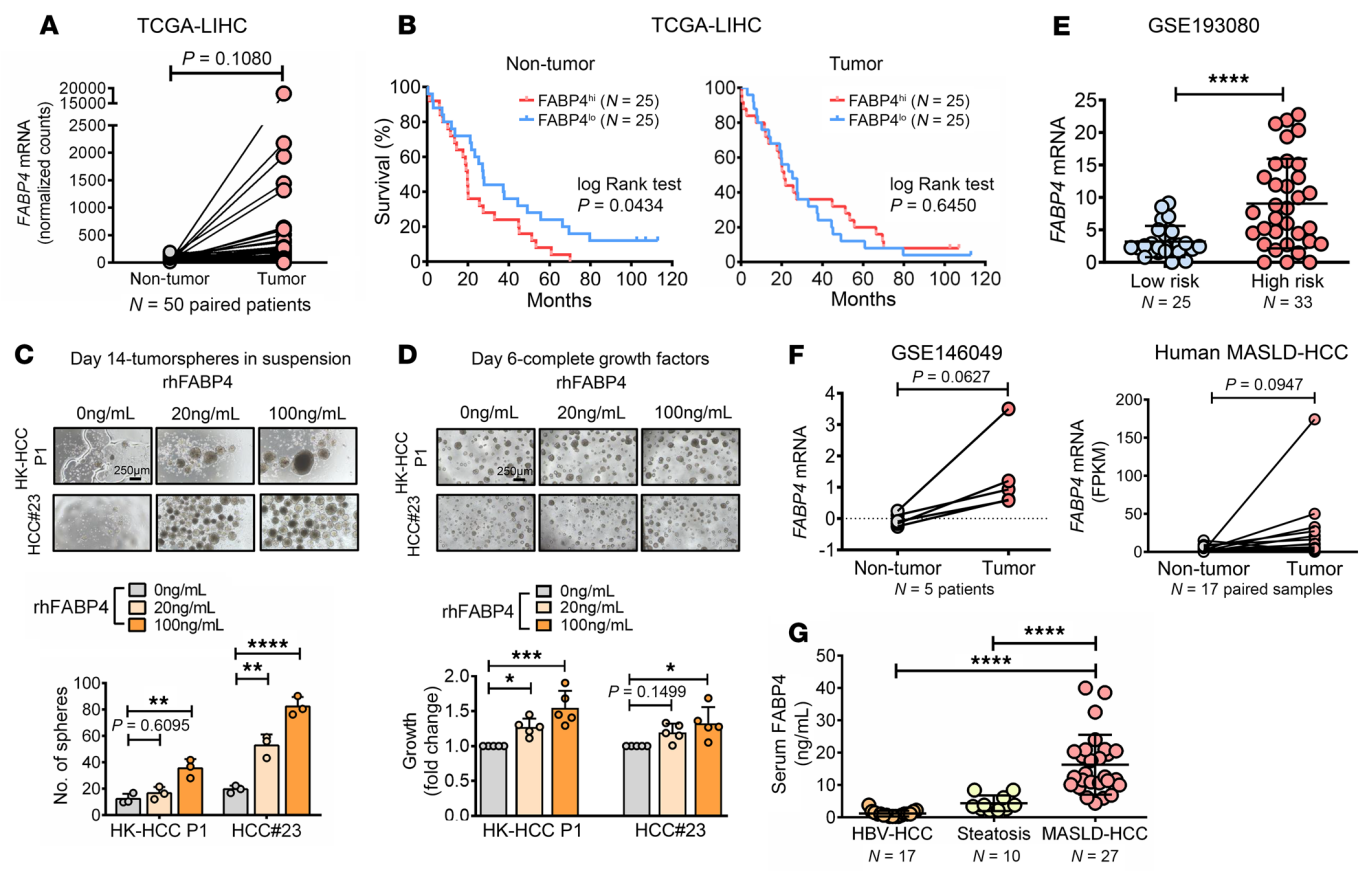
Clinical relevance of FABP4 in HCC. (**A**) *FABP4* expression in paired non-tumor and tumor of HCC patients in the TCGA-LIHC cohort (*P* = 0.1080). (**B**) HCC patients with high *FABP4* had a poorer overall survival rate than those with lower expression in non-tumor in TCGA-LIHC. (**C**) rhFABP4 conferred HCC patient–derived organoids with self-renewal ability (*n* = 3). (**D**) Growth of HCC patient–derived organoids upon administration of rhFABP4 (*n* = 5). Representative images of organoids. Scale bars in **C** and **D**: 250 μm. (**E**) *FABP4* mRNA in patients with MASLD in GSE193080. (**F**) *FABP4* expression in GSE146049 and 17 paired MASLD-HCC of in-house cohort by RNA-seq. (**G**) Serum FABP4 levels in patients with HBV-HCC, steatosis, and MASLD-HCC. Data represent mean ± SD. *P < 0.05, **P < 0.01, ***P < 0.001, ****P < 0.0001. **A**, **E**, **F**: 2-tailed *t* test; **B**: log-rank test; **C**, **D**, **G**: 1-way ANOVA followed by Tukey’s multiple-comparison test.

Analysis of paired tumor and nontumor samples from The Cancer Genome Atlas Liver Hepatocellular Carcinoma (TCGA-LIHC) cohort showed no significant change in FABP4 expression (Figure 4A). However, nontumor samples from the same cohort indicated that patients with high *FABP4* levels had shorter overall survival (Figure 4B). We further examined the effect of rhFABP4 in a more clinically relevant setting — an organotypic ex vivo culture of primary HCC tumor, i.e., HCC patient–derived organoids (HK-HCC P1 and HCC#23). rhFABP4 conferred self-renewal and increased the size of organoids and their proliferative rate in a dose-dependent manner (Figure 4, C and D). Likewise, the secretome in ADCM increased the invasiveness and proliferation rate of HCC organoids (Supplemental Figure 4, A and B). In our in-house MASLD-HCC mouse model, *Fabp4* mRNA levels were significantly upregulated in MASLD-HCC, showing a gradient increase from steatosis to MASH and subsequently to HCC (Supplemental Figure 5). Patients with MASLD exhibiting high *FABP4* expression were at an elevated risk for developing HCC (Figure 4E). Next, we examined the expression level of *FABP4* in patients with MASLD-HCC. *FABP4* was upregulated in MASLD-induced HCC clinical samples compared with their adjacent normal counterparts in a publicly available dataset and our in-house cohort (Figure 4F). Overexpression of *FABP4* in these patients in a different cohort showed a trend of HCC recurrence (GSE214432, *P* = 0.0994, data not shown). To further confirm the specificity of FABP4 in MASLD-HCC, we compared serum samples from patients with steatosis, HBV-associated HCC, and MASLD-induced HCC. Our results indicated that serum FABP4 levels were significantly elevated in MASLD-HCC patients, with a mean level of 16.24 ng/mL, compared with both the HBV-HCC and steatosis groups. This underscores the specificity of FABP4 in MASLD-induced HCC (Figure 4G).

### FABP4 activates the Wnt/β-catenin pathway via phosphorylation of AKT.

**Figure 5:**
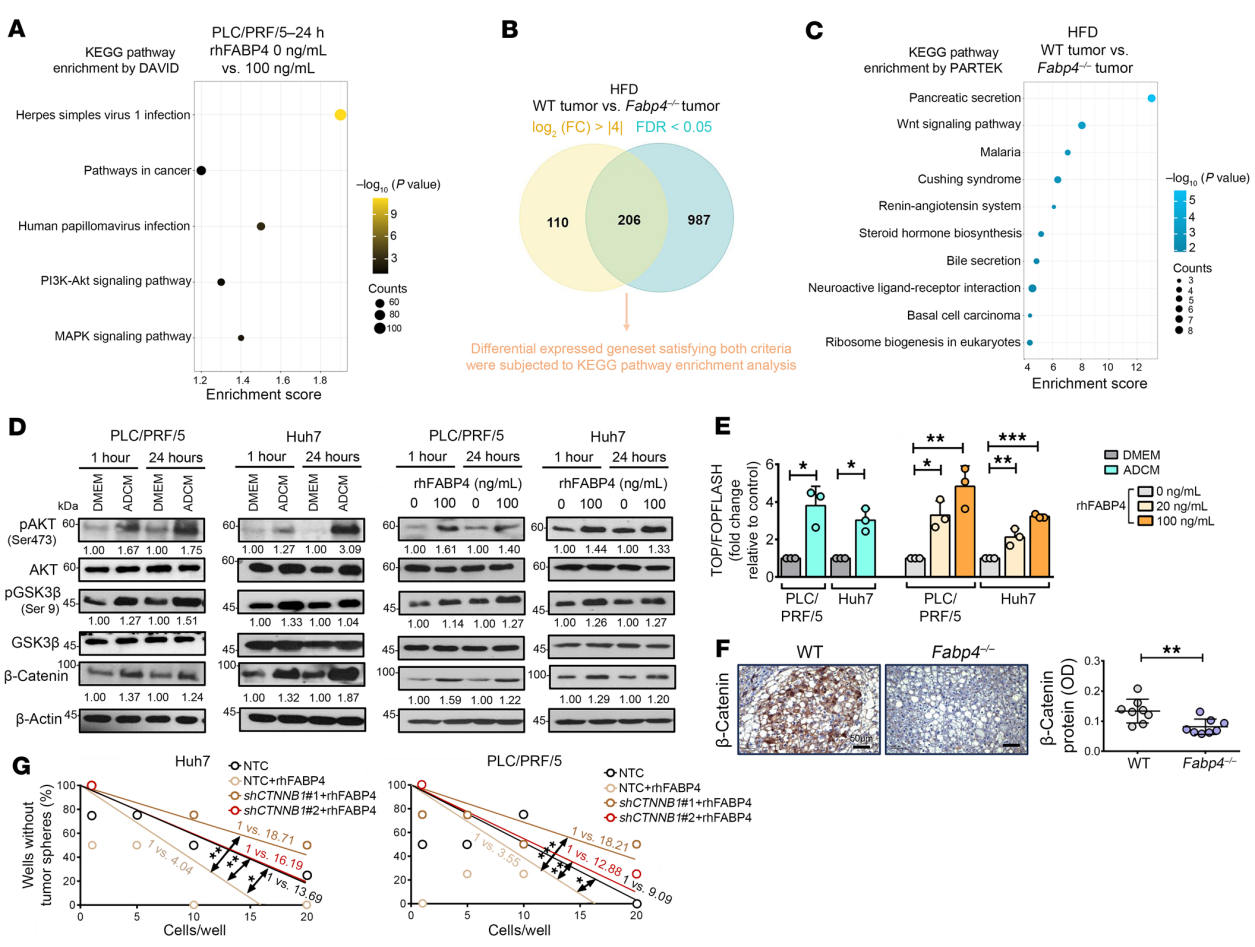
FABP4 activates the PI3K/AKT/Wnt/β-catenin signaling pathway. (**A**) RNA-seq was conducted on PLC/PRF/5 cells treated with rhFABP4. The differentially expressed genes (DEGs) with a fold change >1.5 were subjected to KEGG pathway enrichment. (**B**) Criteria for selection of DEGs upon RNA-seq in tumors from WT and *Fabp4^–/–^* mouse liver after HFD. (**C**) The top 10 enriched pathways were identified based on KEGG pathway enrichment analysis in from WT compared with *Fabp4^–/–^* mouse tumor. (**D**) Western blotting of HCC cells after treatment with either ADCM or rhFABP4. (**E**) Transactivating activity of β-catenin was examined after treatment with either ADCM or rhFABP4 for 24 hours (*n* = 3). (**F**) Immunohistochemical images of β-catenin in resected mouse livers (*n* = 8 mice). Scale bars: 50 μm. (**G**) Limiting dilution sphere analysis showed the role of rhFABP4 in regulation of self-renewal ability upon knockdown of *CTNNB1* (*n* = 2). Data represent mean ± SD. *P < 0.05, **P < 0.01, ***P < 0.001. **E** left, **F**: 2-tailed *t* test; **E** right: 1-way ANOVA followed by Tukey’s multiple-comparison test; **G**: extreme limiting dilution analysis with χ^2^ test.

To elucidate the mechanisms by which FABP4 regulates cancer stemness of HCC cells, we performed bulk RNA-seq profiling using PLC/PRF/5 cells that were pretreated with 0 ng/mL or 100 ng/mL rhFABP4 for 24 hours. Kyoto Encyclopedia of Genes and Genomes (KEGG) pathway enrichment analysis using the Database for Annotation, Visualization, and Integrated Discovery (DAVID; https://davidbioinformatics.nih.gov/) showed that PI3K/AKT signaling was enriched upon treatment with rhFABP4 (Figure 5A). Furthermore, we analyzed the enriched signaling pathways in tumors harvested from WT and *Fabp4^–/–^* mice after HFD feeding based on KEGG pathway enrichment analysis using the Partek Genomics Suite (Illumina) (Figure 5B). Among the top 10 enriched pathways, the Wnt signaling pathway was the only stemness-related pathway enriched in WT tumors (Figure 5C), in which *Ctnnb1* was downregulated in *Fabp4^–/–^* tumors (data not shown). By Western blot analysis, we found that AKT was consistently activated through phosphorylation at Ser473 upon ADCM and rhFABP4 treatment, in turn leading to inactivation of GSK3β via phosphorylation at Ser9, which resulted in accumulation of total β-catenin (Figure 5D). Transactivation of β-catenin was also enhanced, as determined by TOP/FOP reporter assay (Figure 5E). We further confirmed that the protein level of β-catenin was significantly lower in liver tumors of *Fabp4^–/–^* mice (Figure 5F). Last, we confirmed the critical involvement of β-catenin in FABP4-mediated CSC function by suppressing this protein in rhFABP4-treated HCC cells (Figure 5G). The above results suggested that exogenous FABP4 regulates Wnt/β-catenin signaling through activation of PI3K/AKT in HCC cells.

### Exogenous FABP4 directly binds to the membrane receptor ITGB1, driving the PI3K/AKT/β-catenin signaling cascade.

**Figure 6:**
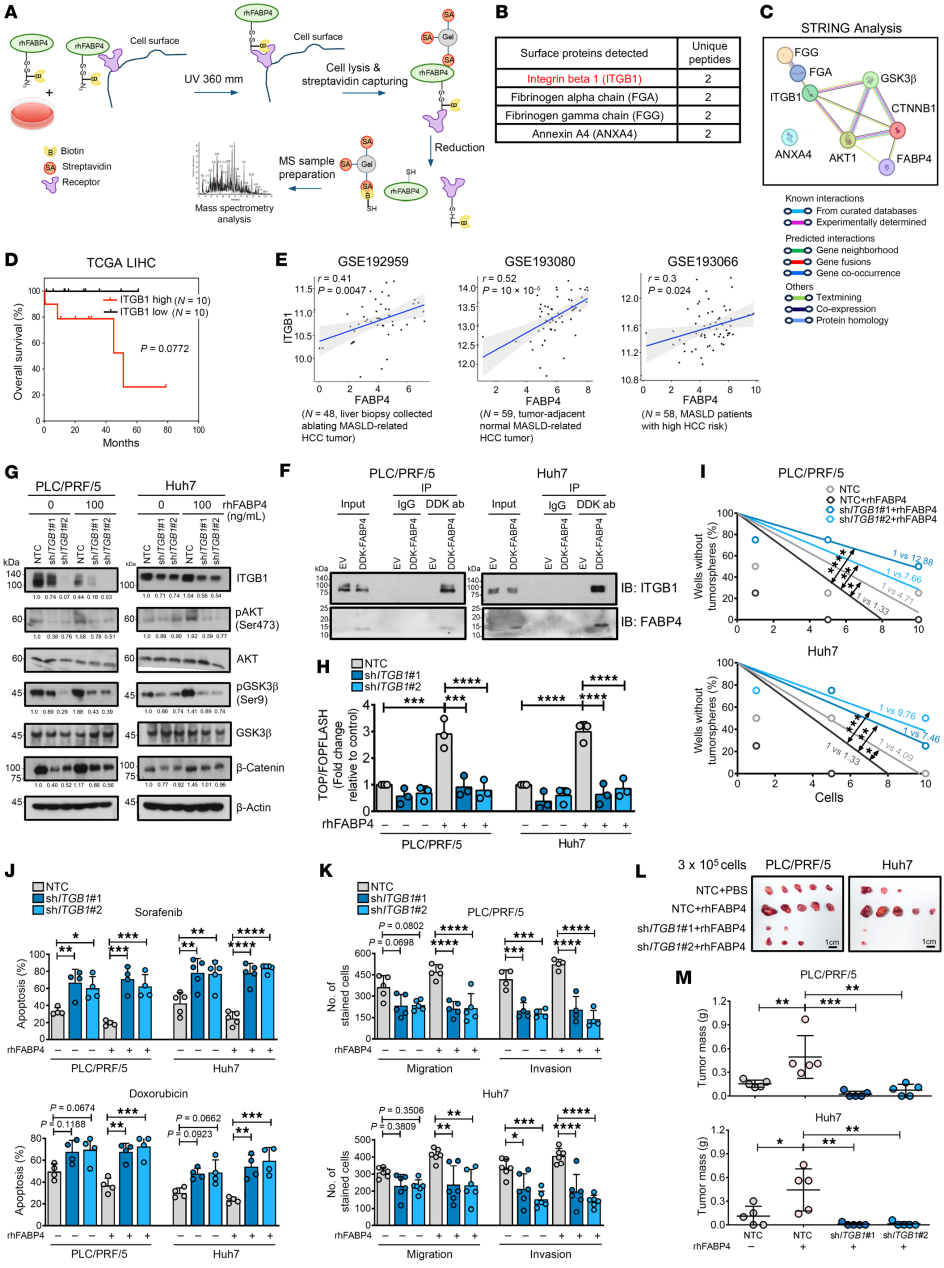
ITGB1 was identified as a receptor of exogenous FABP4 that mediates CSC functions by driving the PI3K/AKT/β-catenin signaling cascade. (**A**) Workflow for analysis of potential FABP4-binding proteins on the membrane surface of Huh7 cells using biotinylated rhFABP4 (*n* = 1). (**B**) Surface proteins detected with ≥2 unique peptides. (**C**) Interactions between potential FABP4-binding proteins and main players in Wnt/β-catenin signaling. (**D**) Among HCC patients with MASLD as risk factor in TCGA-LIHC, those with high *ITGB1* had poorer overall survival rate than those with low *ITGB1* (log-rank test). (**E**) According to GSE192959, GSE193080, and GSE193066, *FABP4* was positively correlated with *ITGB1* in patients with MASLD-related HCC and MASLD patients at high risk of HCC (Pearson’s correlation). (**F**) Reciprocal coimmunoprecipitation demonstrated the interaction between exogenous FABP4 and ITGB1 (*n* = 2). (**G**) Western blot analyses of sh*ITGB1* HCC cells upon rhFABP4 treatment. (**H**) Transactivating activity of β-catenin was examined in sh*ITGB1* HCC cells after treatment with rhFABP4 for 24 hours (*n* = 3). (**I**) Limiting dilution sphere analysis showed the role of ITGB1 in regulation of FABP4-driven self-renewal ability (*n* = 2). NTC, non-target control. (**J**) Apoptosis of *ITGB1*-silenced HCC cells pretreated with PBS (rhFABP4 0 ng/mL), rhFABP4 (100 ng/mL), doxorubicin, or sorafenib for 48 hours. *n* = 4–5. (**K**) Effect of rhFABP4 on cell migration and invasion upon silencing of *ITGB1* (*n* = 4–6). (**L**) *ITGB1*-knockdown and control HCC cells pretreated with either PBS or rhFAPB4 at 100 ng/mL for 24 hours were subcutaneously inoculated into nude mice. Images of xenograft tumors. Scale bars: 1 cm. (**M**) Graphs showing the tumor masses (*n* = 5 mice per group). Data represent mean ± SD. *P < 0.01, **P < 0.01, ***P < 0.001, ****P < 0.0001. **D**: log-rank test; **H**, **J**, **K**, **M**: 1-way ANOVA followed by Tukey’s multiple-comparison test; **I**: extreme limiting dilution analysis with χ^2^ test.

We employed mass spectrometry analysis to identify potential FABP4-binding receptors on the membrane surface of Huh7 cells using biotinylated rhFAPB4 (Figure 6A and Supplemental Figure 6, A–C). In total, 4 surface proteins were detected (Figure 6B). These surface proteins, together with FABP4 and the main canonical components controlling Wnt/β-catenin signaling, were subjected to STRING analysis. ITGB1 potentially interacted with AKT1, GSK3β, and CTNNB1 (β-catenin) (Figure 6C), suggesting that ITGB1 may play a role in the regulatory circuit through which FABP4 mediates the activation of Wnt/β-catenin signaling in HCC. Clinically, in HCC patients with a MASLD background, high *ITGB1* expression was associated with a trend toward shorter overall survival (Figure 6D). Furthermore, we found that *FABP4* expression was positively correlated with *ITGB1* and *CTNNB1* in MASLD-related HCC patients and MASLD patients at high risk of HCC (Figure 6E and Supplemental Figure 7). Consistent with the mass spectrometry findings, the immunoprecipitation data revealed a physical interaction between FABP4 and ITGB1 (Figure 6F). The physical interaction between the FABP4 and ITGB1 complex was further examined using molecular docking analysis. The protein-protein docking result demonstrated that the FABP4-ITGB1 complex was structurally robust (rank-sum score of 458), with a highly complementary binding interface that stabilized by a sophisticated network of high-affinity contacts (Supplemental Figure 6D). Stable knockdown of *ITGB1* in HCC cells led to downregulation of p-AKT (Ser473) and p-GSK3β (Ser9), resulting in a reduction in total β-catenin accumulation and transactivation (Figure 6, G and H). The effects of exogenous FABP4 could be eliminated by repression of ITGB1 (Figure 6, G and H). Furthermore, we found that the enhancing effects of rhFABP4 on self-renewal ability, drug resistance, and migration and invasion capabilities were offset upon the repression of ITGB1 (Figure 6, I–K). Our in vivo observations also showed that suppression of ITGB1 mitigated the effect of rhFABP4-induced HCC tumor growth (Figure 6, L and M). Collectively, these data suggest that ITGB1 is a crucial membrane receptor that mediates cancer stemness and drug resistance in HCC cells via the PI3K/AKT/β-catenin signaling cascade.

### Anti-FABP4 neutralizing mAb suppresses cancer stemness and inhibits tumor growth in a MASLD-HCC mouse model.

**Figure 7:**
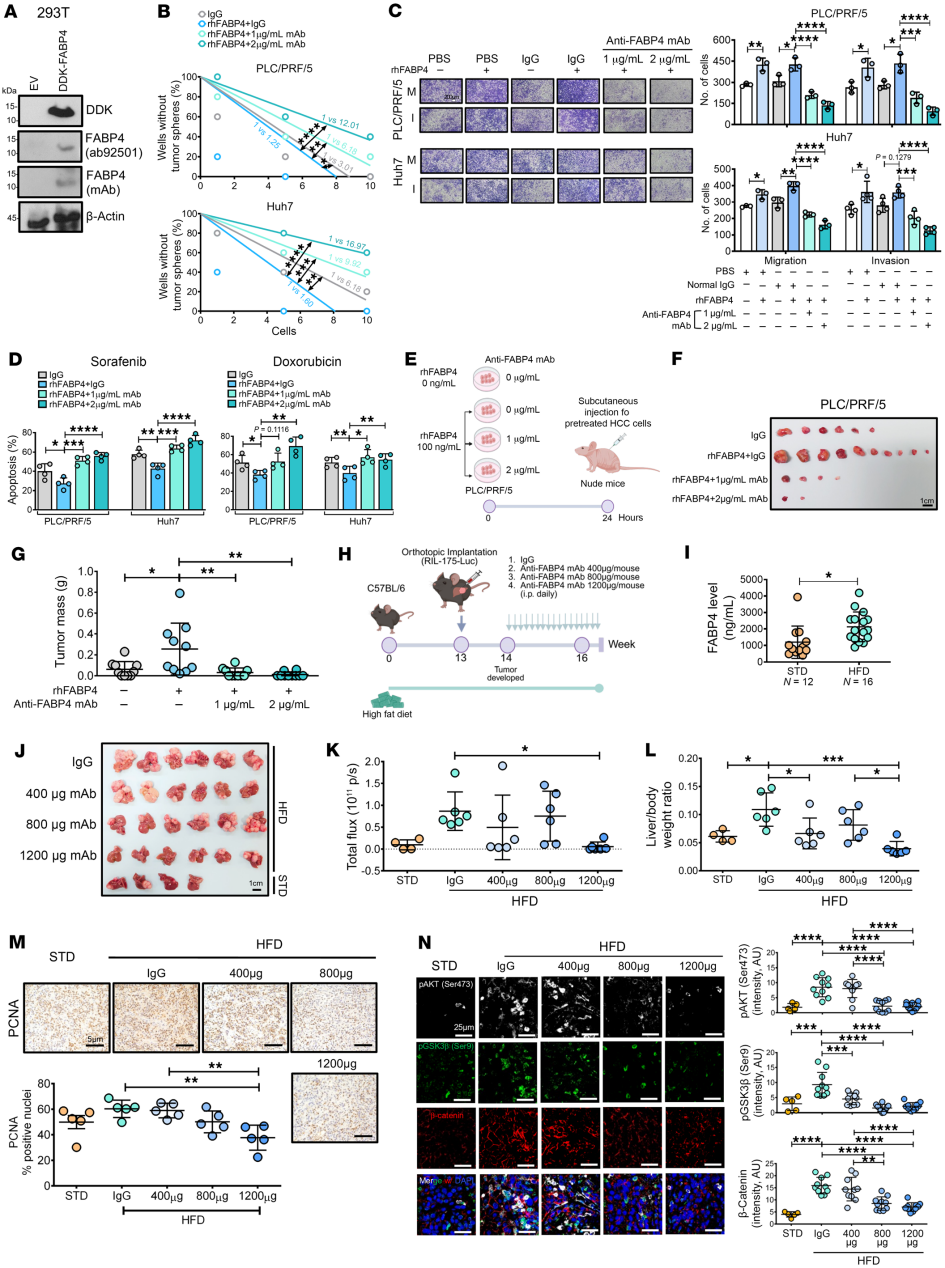
The effect of the anti-FABP4 mAb on CSC properties and tumor suppression in a MASLD-HCC mouse model. (**A**) Western blot showing specificity of FABP4 mAb. (**B**) Limiting dilution sphere analysis of the effect of rhFABP4 on HCC cells following treatment with FABP4 mAb (*n* = 2). (**C**) Effect of ablation of FABP4 on migration (M) and invasion (I) abilities of cells (*n* = 3–4). Representative images of stained cells. Scale bar: 200 μm. (**D**) Apoptosis of HCC cells treated with either PBS (0 ng/mL rhFABP4) or rhFABP4 (100 ng/mL) or in combination with the antiFABP4 mAb induced by doxorubicin or sorafenib for 48 hours (*n* = 4). (**E**) Workflow of pretreatment of HCC cells with rhFABP4 and anti-FABP4 mAb prior to subcutaneous inoculation in nude mice. (**F**) Images of xenograft tumors. Scale bar: 1 cm. (**G**) Graph showing the mass of xenograft tumors (*n* = 10 mice). (**H**) Experimental design for orthotopic MASLD-HCC mouse model with anti-FABP4 mAb treatment. (**I**) Serum FABP4 after 13 weeks of HFD or STD before orthotopic implantation (2-tailed *t* test). (**J**) Image of HCC tumors. Untreated HCC tumors of STD group were included. Scale bar: 1 cm. (**K**) Luciferase signal intensity of livers (STD: *n* = 4 mice and HFD: *n* = 6 mice per group). (**L**) Graph of mouse liver/body weight ratio. (**M**) Immunohistochemical images of PCNA in resected tumors (*n* = 5 random fields). Scale bars: 5 μm. (**N**) Expression of p-AKT (Ser473) (white), p-GSK3β (Ser9) (green), and β-catenin (red) in resected tumors (*n* = 5–10 random fields). Scale bars: 25 μm. **B**: extreme limiting dilution analysis with χ^2^ test; **C**, **D**, **G**, **K**–**N**: 1-way ANOVA followed by Tukey’s multiple-comparison test. DAPI: nuclei, blue. Data represent mean ± SD. *P < 0.05. **P < 0.01, ***P < 0.001, ****P < 0.0001.

We developed a monoclonal antibody targeting FABP4 (anti-FABP4 mAb, 3I19-1) and evaluated its therapeutic potential for MASLD-HCC. Among several ascites samples we generated, ascite 6 showed the greatest neutralizing effect on rhFABP4 in HCC cells and was selected for generation of purified anti-FABP4 mAb (Supplemental Figure 8). The specificity of the antibody was confirmed in DKK-tagged FABP4-overexpressing 293T cells via Western blotting, which revealed a clear band at approximately 12 kDa (Figure 7A). We next sought to examine the neutralizing effect of this anti-FABP4 mAb on FABP4-driven cancer stemness. The anti-FABP4 mAb effectively abolished the ability of rhFABP4 to enhance self-renewal, cell migration and invasion, and drug resistance (Figure 7, B–D). Furthermore, the neutralizing effect of the anti-FABP4 mAb on rhFABP4-induced tumor incidence rate and tumor growth was also evidenced in PLC/PRF/5 cells when these cells were cotreated with 100 ng/mL rhFABP4 prior to subcutaneous injection into nude mice (Figure 7, E–G). Next, we investigated the therapeutic potential of the anti-FABP4 mAb in suppressing tumor growth in a MASLD-HCC mouse model induced by orthotopic injection of RIL-175 mouse HCC cells into the liver of mice fed a HFD for 13 weeks (Figure 7H). We observed a drastic increase in the serum FABP4 level in the HFD group compared with the standard diet (STD) group (Figure 7I). After tumor development 1 week after implantation, anti-FABP4 mAbs were administered, at 400 μg, 800 μg, and 1,200 μg, via i.p. injection. After treatment for 17 days, 1,200 μg of the anti-FABP4 mAb effectively suppressed tumor growth (Figure 7, J–L). During this experiment, no obvious loss of body weight was observed in the animals (Supplemental Figure 9). Upon treatment, HCC cells showed a decreased number of proliferating cell nuclear antigen–positive (PCNA-positive) nuclei (Figure 7M). Additionally, expression levels of p-AKT (Ser473), p-GSK3b (Ser9), and β-catenin began to decrease with the introduction of anti-FABP4 antibody at concentrations as low as 400 μg (Figure 7N). Furthermore, FABP4 neutralization mitigated the steatotic and inflammatory states, as evidenced by decreased lipid accumulation, inhibition of inflammatory gene expression, and a reduced M1/M2 macrophage ratio (Supplemental Figure 10, A–D). Echoing the findings in the DEN-HFD MASLD-HCC mouse model, FABP4 was also found to be colocalized with lipid droplet–enriched steatotic hepatocytes (Supplemental Figure 10E). Taken together, these findings indicate that targeting adipocyte-derived FABP4 signaling pathway is a promising effective treatment for MASLD-related HCC.

## Discussion

In this study, we revealed that adipocytes regulate liver CSC functions via paracrine secretion and that this effect is further enhanced when adipocytes are cocultured with HCC cells. This result is consistent with a previous report showing the role of adipocytes in promoting tumor growth, metastasis, and drug resistance in ovarian cancer (28, 29). In MASLD, steatosis triggers inflammation by recruiting and activating macrophages. The chronic inflammatory state leads to the progression of this disease to HCC (30). Notably, when differentiated macrophages derived from THP1 were exposed to ADCM-HCC CM, these stimulated macrophages were observed to contribute to increased sphere formation, indicative of an increase in cancer stemness (data not shown). Nonetheless, the enhancing effect conferred by stimulated adipocytes was more pronounced. This finding further reinforces the indispensable role of adipocytes in conferring CSC properties to HCC cells. Mass spectrometry revealed that FABP4 was enriched in adipocytes cocultured with HCC cells, and we also detected a substantial amount of FABP4 in the serum of patients with MASLD-HCC. Our result is in line with previous reports showing increased levels of circulating FABP4 in individuals with obesity (31) and in individuals with decompensated cirrhosis (32). In addition, MASLD patients showed a substantial increase in serum FABP4 levels compared with those without liver disease (33, 34), and this upregulation persisted in MASLD-HCC patients (34). Our data from patients align with previous findings showing increased serum FABP4 in MASLD-HCC compared with HBV-induced HCC patients (34). Notably, serum FABP4 levels positively correlated with BMI in MASLD-HCC patients (Pearson’s *r* = 0.4467), while the correlation was weak in HBV-induced HCC patients (Pearson’s *r* = 0.0701). This suggests that adiposity contributes to elevated FABP4 levels. Elevated levels of serum FABP4 have been reported not only in MASLD-HCC patients but also in obesity-associated breast cancer patients compared with nonobese patients (35).

Circulating FABP4 promoted obesity-associated breast cancer by increasing mammary tumor stemness and aggressiveness through the IL-6/STAT3/ALDH1 axis (31). Subsequently, it has been reported that FABP4 depletion suppresses the activation of stemness properties in colorectal cancer via modulation of the ERK/mTOR pathway (36). Although a role for FABP4 in the regulation of cancer stemness has been suggested, the functional and mechanistic roles of adipose-derived FABP4 in cancer stemness remain unknown. By exogenously administering rhFABP4, we demonstrated that FABP4 was critically involved in the regulation of liver CSCs. These data, together with the suppression of the CSC-enhancing effect of adipocytes by BMS-309403, suggest that adipose-derived FABP4 plays a role in the regulation of liver CSCs. In addition, FABP4 also conferred resistance to doxorubicin and sorafenib, which is consistent with other findings showing the role of FABP4 in driving drug resistance in ovarian cancer (29). Using *Fabp4^–/–^* mice, we first demonstrated the critical oncogenic role of FABP4 in MASLD-induced HCC. The delayed oncogenic effect may have been due to the loss of FABP4 in liver as well as the distinct visceral adipocytes. However, given that the knockout was global, it does not provide specific elucidation of the interactions between adipocytes and liver cells. Interestingly, we observed decreased inflammation and reduced fibrosis in *Fabp4^–/–^* mice. This finding is consistent with previous reports demonstrating attenuation of fibrosis in chemically induced liver fibrosis models (37) and reduced inflammation in liver injury mouse models (38). The finding suggests that these intrinsic factors also hinder HCC tumor formation, in addition to the paracrine effects of FABP4. While our current study does not directly address the local effects of hepatic FABP4, we believe we have comprehensively demonstrated the impact of adipose tissue–derived FABP4 on HCC cells, providing insights into HCC pathogenesis, particularly within the context of obesity.

Bulk RNA-seq analysis revealed that exogenous FABP4 regulated liver cancer stemness through the PI3K/AKT/β-catenin signaling pathway. As a secretory factor, identifying the receptor mediating this pathway is crucial. Only one study has shown CD36 as a direct binding partner of FABP4, facilitating fatty acid transfer from adipocytes to breast cancer cells (39). We investigated CD36 suppression’s role in modulating β-catenin signaling but the results were inconclusive. Therefore, we used streptavidin capture-based mass spectrometry with biotin-labeled rhFABP4 to identify possible membrane binding targets of FABP4 in HCC cells. Our analysis identified ITGB1 as a membrane receptor that directly interacted with rhFABP4, as confirmed by reciprocal coimmunoprecipitation analysis. Furthermore, molecular docking analysis revealed that LYS-105 and GLY-67 of FABP4 formed strong hydrogen bonds with ASP-687 and ARG-693 of ITGB1 at distances of 2.6 Å and 2.3 Å, respectively. Additionally, LYS-100 and GLY-99 of FABP4 interacted with TYR-712 and GLU-710 of ITGB1 at distances of 3.2 Å and 2.2 Å, respectively. This precise spatial configuration of hydrogen bonds highlights a highly selective molecular recognition mechanism, optimizing both binding affinity and structural stability. ITGB1 was found to be overexpressed in cancers such as gastric (40) and breast (41) cancer, correlating with poorer clinical outcomes (40, 41). Analysis of the TCGA-LIHC cohort showed that HCC patients with MASLD, a condition linked to high *ITGB1* expression, had worse overall survival compared with those with low *ITGB1* expression. ITGB1 promotes cell proliferation and migration in colorectal cancer (42) and sensitizes HCC cells to sorafenib treatment upon its ablation (43). Our results indicated ITGB1’s crucial role in linking FABP4 with the AKT/β-catenin signaling pathway, evidenced by inhibited CSC-promoting and AKT/β-catenin signaling in ITGB1-suppressed cells with rhFABP4 addition. Analysis of datasets from MASLD-HCC patients and from MASLD patients with high HCC risk revealed a significant positive correlation between FABP4 and either ITGB1 or CTNNB1, supporting ITGB1’s role in adipocyte-derived FABP4-driven MASLD-HCC.

Strategies targeting FABP4 have been previously reported to treat acute liver injury and nonalcoholic steatohepatitis in mice via the pharmacological inhibitor BMS-309403 (38). However, this drug induced an acute cardiac depressant effect, which limits its clinical use (44). In addition to the inhibitor approach, several studies blocking the function of FABP4 with an antibody have reported encouraging results. Treatment with the FABP4 monoclonal antibody CA33 exhibits an antidiabetic effect in HFD-induced obese mice, with a reduction of fat mass (45). Recently, FABP4 mAb 6H2 effectively blocked FABP4-induced cerebral ischemia injury (46). However, there are no studies showing the therapeutic effect of FABP4 mAb in HCC, particularly MASLD-HCC. We developed an anti-FABP4 mAb that not only demonstrated the specificity but also, and most importantly, attenuated the CSC-enhancing effects of rhFABP4, together with the suppression of the AKT/β-catenin pathway. Pretreatment with the FABP4 mAb in HCC cells also successfully impeded the rhFABP4-driven tumorigenic effect. Intriguingly, administration of 1,200 μg mAb for 17 days effectively suppressed tumor growth in an orthotopic MASLD-HCC mouse model, which was accompanied by suppression of the AKT/β-catenin signaling pathway. Furthermore, we demonstrated that blockage of FABP4 action through either genetic ablation or neutralization approaches led to suppression of steatosis and inflammation, which may potentially attenuate HCC tumor growth.

In conclusion, we demonstrated the crucial role of adipocyte-derived FABP4 in the regulation of cancer stemness in HCC, and its effect was enhanced by coculture with HCC cells. Our results indicate that the FABP4-ITGB1-AKT/β-catenin pathway is a key signaling cascade driving MASLD-related HCC. Targeting secretory FABP4 with a FABP4 mAb impeded the growth of MASLD-HCC tumors, suggesting a potential therapeutic avenue for the treatment of this deadly disease.

## Methods

### Sex as a biological variable.

Our study examined samples from both male and female donors. Experiments were conducted on female and male mice in separate studies. Sex was not considered as a biological variable. While our study focused on male mice, the findings may be relevant for both sexes.

### Cell lines and cell culture.

The following cells lines were maintained in DMEM with high glucose and l-glutamine (Gibco, Invitrogen) supplemented with 10% heat-inactivated FBS (Gibco, Invitrogen), 100 mg/mL penicillin G, and 50 μg/mL streptomycin (Gibco, Invitrogen) at 37°C in a humidified chamber containing 5% CO_2_: HSC-hTERT (ATCC); Huh7 and PLC/PRF/5 human HCC cell lines (Japan Cancer Research Bank), and 293FT (Invitrogen, Thermo Fisher Scientific). The culture medium was refreshed every 2 days. Human visceral preadipocytes (PT5005, Lonza) were maintained in preadipocyte basal medium supplemented with FBS, l-glutamine, gentamicin sulfate, and amphotericin-B from Lonza. Preadipocytes were differentiated into adipocytes using preadipocyte basal medium supplemented with recombinant human insulin, dexamethasone, indomethacin, and IBMX according to manufacturer’s protocol. Morphological changes in adipocytes were observed within a week of differentiation, and incubation with differentiation medium for 20 days resulted in 70% differentiation of preadipocytes to adipocytes with lipid accumulation. All cell lines used in this study were obtained between 2013 and 2016, and they were authenticated by morphological observation and STR DNA analysis with PowerPlex *16HS kit* (Promega) as well as tested for the absence of mycoplasma contamination (MycoAlert, Lonza). Cells were used within 20 passages after thawing.

### Patient samples.

Venous blood samples were collected from 27 individuals diagnosed with MASLD-related HCC before any therapeutic interventions and 10 individuals diagnosed with steatosis. The use of human samples was approved by the ethics committee for research involving human subjects at the School of Public Health, Sun Yat Sen University. Seventeen HCC patients with hepatitis B virus infection were recruited from the Comprehensive Liver Cancer Center, the Fifth Medical Center of the PLC General Hospital, and venous blood samples collected as hepatitis virus induced HCC group. This study was approved by the Institutional Review Board for ethical review. Tumors and paired nontumor liver tissues from 17 patients diagnosed with HCC and MASH were obtained from Prince of Wales Hospital, The Chinese University of Hong Kong, and Zhongshan Hospital of Fudan University. This study was approved by the ethics committee of the Chinese University of Hong Kong and the clinical research ethics committee of Zhongshan Hospital of Fudan University. All the above samples were obtained with the written informed consent of the patients.

### Flow cytometric analysis.

Cells were stained by phycoerythrin-conjugated (PE-conjugated) CD47 and CD90 (#536046 and #555596), CD133 (#130-080-901, Miltenyi Biotec), and FITC-conjugated CD44 (#554478 BD Biosciences) antibodies in PBS with 2% FBS at 4°C for 30–60 minutes. Isotype-matched mouse immunoglobulins served as controls. Samples were analyzed using BD Accuri C6 flow cytometer and FACSDiva software (BD Biosciences).

### FABP4 mAb production.

Anti-FABP4 mAb was synthesized using epitope peptide GQEFDEVTADDRKV (aa 68-81 of human FABP4), which targets the ligand binding region of FABP4 (AbMart). US nonprovisional patent (application no. 19/184,090) for the use of FABP4 monoclonal antibody (3I19-1) as a treatment for MASLD-induced HCC has been filed.

### Development of MASLD-HCC model.

*Fabp4*-KO mice in a C57BL/6N background were generated using the same procedures as previously described (46). Age-matched male *Fabp4*-KO mice and their littermates were used in this study. Animals were allocated to their experimental group according to their genotypes. The investigators were not masked to the experimental groups. For the chemically induced MASLD-HCC model, male WT C57BL/6N and *Fabp4*-KO mice were treated with DEN at 25 mg/kg i.p. (Sigma-Aldrich) at the age of 14 days. Starting at 6 weeks of age, they were fed a HFD (D12492, Research Diets) for 29 weeks. Serum and liver samples of mice were collected for analysis at the end point. Tumor nodules in the livers were counted by visual inspection.

### The effect of FABP4 antibody in the MASLD-HCC model.

An orthotopic MASLD-HCC mouse model was established using female C57BL/6J mice (The Jackson Laboratory) provided by Centralized Animal Facility of The Hong Kong Polytechnic University. HFD was provided to the mice at 4 weeks of age for 13 weeks prior to tumor cell inoculation. Seven thousand five hundred luciferase-labeled mouse HCC RIL-175 cells, resuspended in 50% Matrigel, were orthotopically injected at the left lobes of the livers. Tumor growth was monitored by the IVIS imaging system (Perkin-Elmer). Either isotypic IgG control or FABP4 mAb at 400 μg, 800 μg, and 1,200 μg per mouse was injected i.p. once daily for 17 days. The STD (#5053, Rodent Diet 20, PicoLab) group was set up as control. In addition, PLC/PRF/5 cells were pretreated with 100 ng/mL rhFABP4 and either 1 μg/mL or 2 μg/mL anti-FABP4 mAb for 24 hours prior to subcutaneous inoculation in nude mice. The study protocol was approved by and performed in accordance with the guidelines for the Use of Live Animals in Teaching and Research at The Hong Kong Polytechnic University. Mice were sacrificed if the percentage of body weight loss was greater than 20%, including the tumor mass. The maximal tumor size/burden was permitted by the study protocol of The Hong Kong Polytechnic University. The maximal tumor size/burden was not exceeded in any experiments.

### Steatosis and MASLD-HCC mouse models.

Male C57BL/6 WT littermates (8 weeks old) were fed a normal chow (NC) diet, high-fat low-cholesterol diet (HFLC), or high-fat high-cholesterol diet (HFHC) (Specialty Feeds) ad libitum for 14 months. Mice fed HFHC developed MASLD-HCC, while HFLC diet induced only steatosis.

### Statistics.

The statistical significance of the results obtained from limiting dilution assay, flow cytometric analysis, migration and invasion assay, qRT-PCR, immunohistochemical and immunofluorescence staining, and in vivo tumor growth and volume analysis were determined by 2-tailed *t* test wherever appropriate using GraphPad Prism (GraphPad Software). One way ANOVA was applied wherever appropriate. The results are presented as mean and SD, and *P* values less than 0.05 were considered statistically significant. A data point was excluded if it deviated from the mean by more than 3 SDs. Investigators were not masked to the group allocation during the experiment or when assessing the outcome in any experiment, including animal experiments. There was no estimate of variation within each group of data. The variance was similar between the groups that were being statistically compared. Kaplan-Meier survival analysis was used to analyze overall survival, and a log-rank test was used to determine statistical significance.

### Study approval.

Human MASLD-HCC tumor tissues and adjacent normal tissues were collected in Prince of Wales Hospital, The Chinese University of Hong Kong, from donors with biopsy-proven MASLD-HCC (*n* = 17). Written informed consent was obtained from all participants, and the study protocol was approved by the Clinical Research Ethics Committee of The Chinese University of Hong Kong and the University of Hong Kong. Venous blood samples were collected from 27 patients with MASLD-related HCC before any therapeutic procedures were performed. Use of human samples was approved by the committee for ethical review of research involving human subjects at School of Public Health, Sun Yat Sen University.

### Data availability.

Bulk RNA-seq data for PLC/PRF/5 cells, untreated (rhFABP4_0ng) and treated with rhFABP4 at 100 ng/mL (rhFABP4_100ng), as well as HCC tumors derived from WT and *Fabp4*-KO mice, have been deposited in the NCBI’s Gene Expression Omnibus (GEO GSE292914 and GSE286130, respectively). All individual values represented in graphs are provided in the Supporting Data Values file.

## Author contributions

CONL, SG, KPSC, and TKWL conceptualized the study. CONL, SG, KPSC, RWHL, MMLL, MSMC, GKM, SAK, and XPW devised the methodology. CONL, SG, KPSC, SM, and TKWL analyzed the data. CONL and TKWL wrote the original draft. SM, JY, HLZ, XQW, YYL, and RLCH provided reagents and human samples for this study. CONL and TKWL reviewed and edited the manuscript.

## Funding support

- National Natural Science Foundation of China, 82073275.
- Innovation and Technology Fund, MRP/043/21 and ITS/042/23.
- RGC Collaborative Research Fund, C7026-18G.
- RGC Research Impact Fund, R7022-20 and R5008-22F.

## Supplementary Material

Supplemental data

Unedited blot and gel images

Supporting data values

## Version 1 12/15/2025

Electronic publication
