# Supplementary material for: Adipocyte-derived FABP4 promotes metabolism-associated steatotic liver–induced hepatocellular carcinoma by driving ITGB1-mediated β-catenin activation
Source: J Clin Invest. 2025 Dec 15;135(24):e182322. doi: 10.1172/JCI182322 (PMC12700556; doi:10.1172/JCI182322)
Supplement: Supplemental data [file jci-135-182322-s277.pdf]

# Adipocyte-derived FABP4 promotes metabolism-associated steatotic liver-induced hepatocellular carcinoma by driving ITGB1-mediated β-catenin activation

Carmen Oi Ning Leung, Shilpa Gurung, Katherine Po Sin Chung, Rainbow Wing Hei Leung, Martina Mang Leng Lei, Mandy Sze Man Chan, Gregory Kenneth Muliawan, Shakeel Ahmad Khan, Xue Qian Wu, Jun Yu, Hui Lian Zhu, Yin Ying Lu, Stephanie Ma, Xiao Ping Wu, Ruby Lai Chong Hoo, Terence Kin Wah Lee

Table of contents

Supplemental methods……………………………………………………………………………..…2

Supplemental figures………………………………………………………………………………….14

Supplemental tables……………………………………………………………………………..…24

Supplemental references……………………………………………………………………..…….29

**Supplemental methods**

*Reagents.* The recombinant human FABP4 (rhFABP4) protein was purchased from BioVision. The FABP4 inhibitor, BMS-309403, was purchased from Cayman.

*Assessment of steatosis and lobular inflammation scores.* The steatosis and lobular inflammation scores of the mouse livers were assessed using Hematoxylin and Eosin (H&E) stainings. Steatosis was scored according to the following criteria validated by the Pathology Committee of the NASH Clinical Research Network [1]: 0 (<5%), 1 (5-33%), 2 (>33-66%) or 3 (>66%) of parenchymal involvement of steatosis. Inflammation was scored by overall assessment of all inflammatory foci according to the following criteria: 0 (No foci), 1 (< 2 foci per field), 2 (2-4 foci per field) or 3 (> 4 foci per field). Five random fields were assessed in each sample.

*HCC patient-derived organoids and culture conditions.* HCC tissues were obtained from patients undergoing hepatectomy at Queen Mary Hospital, Hong Kong. Samples were collected from patients who had not received any previous local or systemic treatment prior to operation. Informed consent was obtained from all patients before the collection of liver specimens and the study was approved by the Ethics Committee of the University of Hong Kong. For organoid cultures, cells were isolated and cultured according to published protocol [2]. HCC patient-derived organoids labelled HCC#23 was gift from Dr. Meritxell Huch of The Gudon Institute at the University of Cambridge.

*Oil Red-O staining*. Preadipocytes and adipocytes seeded on coverslips were fixed in 4% paraformaldehyde (PFA) for 10 minutes prior to twice of PBS washings. The fixed cells were

incubated with isopropanol for 5 minutes and stained with Oil Red-O solution (Sigma Aldrich) for 15 minutes. Cells were counterstained with hematoxylin for 5 minutes. The stained cells were washed twice with PBS and subjected to microscopic examination.

*Picro sirius red staining.* Paraffin embedded mouse liver tissues were dewaxed and rehydrated. The tissues were stained with 0.1% Picro Sirius red solution for 1 hour, washed twice with 0.5% acidified water. The stained tissues were dehydrated in absolute ethanol, cleared with xylene and mounted for examination. The signal intensity was quantified using ImageJ software.

*BODIPY$^{TM}$ 493/503 staining for neutral lipid droplets.* Frozen mouse liver tissues were fixed with 4% PFA for 10 minutes following twice of PBS washings. The neutral lipid droplets were stained using BODIPY$^{TM}$ 493/503 (Invitrogen, D3922) at 1 μg/mL for 1 hour at 37 ℃. Cells were washed with PBS. Slides were counterstained with DAPI for nuclei, mounted and subjected to Leica TCS SPE confocal microscope examination. The fluorescent intensity of lipid droplets was qualified using ImageJ.

*Collection of conditioned media (CM).* Preadipocytes, adipocytes, HSC-hTERT and HCC cells were seeded in 6-well plate until 80% confluency was attained. Respective culture media were removed and 1.5 mL serum free media was added per well for incubation of 72 hours at 37 ℃. The CM was centrifuged at 3,000 rpm at 4 ℃ for 5 minutes and supernatant was collected.

*Mass spectrometry analysis of CM*. To identify the possible components of the secretomes of the adipocytes, HCC-stimulated adipocyte CM (CAACM), HCC CM (HCCCM) and DMEM (as control), the CM obtained after incubating with respective conditions in serum free medium for 72 hours were collected. The CM was centrifuged at 3,000 rpm for 5 minutes. One milliliter of CM was collected, concentrated and buffer exchanged. Samples were supplemented with urea and trypsin digestion was performed overnight at 37℃. Samples were desalted using C18 columns (Thermo Fisher Scientific). Fractions were eluted using acetonitrile and 0.1% trifluoroacetic acid. Eluted samples were dried, reconstituted with 0.1% formic acid and subjected for Orbitrap mass spectrometry analyses.

*Knockdown of ITGB1 and CTNNB1.* To establish ITGB1 and CTNNB1 knockdown clones, lentiviral particles were generated by co-transfecting 293FT cells with sh*ITGB1* or sh*CTNNB1* cloned using pLKO.1-puro lentiviral vector or non-target control (NTC) plasmids and packaging plasmid mix. Viral supernatant was collected for infection of PLC/PRF/5 and Huh7. The shRNA sequences were listed in **Supplemental Table 4**.

*Cell viability assay.* Cell viability of organoid cultures treated with specified concentrations of rhFABP4 for 6 days was evaluated using CellTiter-Glo® Luminescent Cell Viability Assay (Promega) according to manufacturer's protocol.

*Sphere formation assay.* Three to five hundred cells of PLC/PRF/5, Huh7 and HCC organoids (HK-HCC P1 and HCC#23) were seeded to 24-well plate coated with poly (2-hydroxyethyl methacrylate) (polyHEMA, Sigma Aldrich). Cells were grown in 500 µl of either conditioned media or serum-free 0.25% methylcellulose (Sigma-Aldrich) DMEM/F12 medium

supplemented with 4 μg/mL insulin (Sigma-Aldrich), B27 (Invitrogen) and recombinant human FABP4 (rhFABP4, Biovision) at 20 ng/mL or 100 ng/mL. The number of tumorspheres (diameter larger than 100μm) was counted under microscope after 8-12 days.

For sphere formation assay using co-culturing system, $4x10^4$ adipocytes or preadipocytes were seeded into the insert with pore size of 0.4 μm (Merck Millipore, Germany) while 300-500 HCC cells were seeded in the lower chamber in 500 µl serum-free 0.25% methylcellulose (Sigma-Aldrich) DMEM/F12 medium. The number of tumorspheres was counted after 10 days.

*Limiting dilution assay*. Parental, sh*ITGB1* or sh*CTNNB1* PLC/PRF/5 and Huh7 cells were cultured in a 96-well plate coated with polyHEMA with 200 µl serum-free 0.25% methylcellulose DMEM/F12 medium supplemented with 4 μg/mL insulin, B27 and rhFABP4 when appropriate. Sphere formation was scored after 8-12 days under a phase contrast microscope. The frequency of sphere-forming cell was calculated accordingly using an extreme limiting dilution algorithm (ELDA) (http://bioinf.wehi.edu.au/software/elda/) [3].

*Migration and invasion assays.* Migration assays were performed using polycarbonate membrane transwell inserts with pore size of 8 μm (Millipore). Invasion assays were performed using self-coated Matrigel (BD Biosciences) transwell inserts. HCC cell lines (PLC/PRF/5 and Huh7) or patient derived organoids (HK-HCC P1 and HCC#23) in serum-free medium were seeded in upper chamber while the lower chamber was supplemented with either ADCM or 100 ng/mL rhFABP4 in serum free medium as chemoattractant. Serum free DMEM was used as control. Cells were incubated in humidified incubation at 37 °C for 48

hours. The transwell membranes were fixed with methanol and stained with 1% crystal violet. The membranes were cleaned and air-dried. Photographs of five randomly selected fields of the fixed cells were captured and the cells were counted using ImageJ.

*Enzyme linked immunosorbent assay (ELISA).* Serum FABP4 from patients and secretory FABP4 in conditioned media in human cell lines was quantified using the human FABP4 Duo Set ELISA kit (DY3150-05, R&D Systems). FABP4 and AFP levels in mouse sera were quantified using mouse FABP4 ELISA kit (RD291036200R, BioVendor) and mouse AFP Quantikine ELISA kit (MAFP00, R&D Systems). Quantitation was performed according to manufacturers' protocols.

*Biotinylation of rhFABP4 and Streptavidin capturing of membrane protein.* rhFABP4 was biotin-labelled and crosslinked with sulfosuccinimidyl-2-[6-(biotinamido)-2-(*p*-azidobenzamido) hyxanoamido]ethyl-1,3'-ithiopropionate) (Sulfo-SBED, A39260, Thermo Fisher Scientific) according to manufacturer's instructions. In brief, 50 µg of rhFABP4 (#4504, BioVision) was incubated with 1 mg of dissolved Sulfo-SBED at room temperature for 30 minutes. The reaction mixture was desalted using Amicon® Ultra-0.5 centrifugal filter device with 3,000 Molecular Weight Cut Off (Millipore). The desalted biotinylated rhFABP4 was incubated in PBS with adherent Huh7 cells at room temperature for 10 minutes. The cell surface proteins interacting with biotinylated rhFAPB4 was captured by the photoreactive aryl azide moiety using long wave-UV at 360 nm. The labeled proteins were isolated by NeutrAvidin Agarose slurry and reduced using Pierce™ Cell Surface Protein Biotinylation and Isolation Kit (#A44390, Thermo Fisher Scientific). The interacting rhFABP4-surface protein complex was isolated and the disulfide bond reduced resulting in the biotin label being transferred to the interacting proteins.

*Liquid Chromatography with tandem mass spectrometry (LC-MS/MS) analysis.* The purified samples were prepared for mass spectrometry using EasyPep$^{TM}$ Mini MS Sample Prep Kit (#A40006, Thermo Fisher Scientific) according to manufacturer's protocol. Eluted peptides were subjected to LC-MS/MS analyses at University Research Facility in Chemical and Environmental Analysis in The Hong Kong Polytechnic University, which were performed on Orbitrap Fusion Lumos Mass Spectrometer (Thermo Fisher Scientific) coupled with a Dionex UltiMate$^{TM}$ 3000 RSLnano system (Thermo Fisher Scientific). Peptides were first trapped with a 1mm i.d. × 5mm length trap cartridge (Thermo Fisher Scientific) for 10 min with trapping flow of 10 µL/min, and was then separated on a PepMap C18 column (75 µm i.d. × 25 cm length) with 2 µm particle size (Thermo Fisher Scientific). Mobile phases A and B consisted of 0.1% FA in water and 0.1% FA in ACN, respectively. The LC gradient for separation with a flow rate of 300 nL/minute is as follow: mobile phase B at 6% for 12 minutes; mobile phase B was increased to 20% at 82 minutes and 30% at 92 minutes, followed by 90% mobile phase B at 100 minutes and held for 5 minutes; and mobile phase B was returned to 2% at 105 minutes and maintained until 120 minutes. Both trapping and separation are performed in a column oven at 50$^{o}$C. Data was collected in data dependent acquisition (DDA) mode. The precursor ions with a charge state of 2+ or higher were fragmented by higher-energy collisional dissociation (HCD) with normalized collision energy of 30%. The MS1 Orbitrap resolution was set at 60,000 with standard AGC target in the scan range of 400-1500 m/z and maximum injection time of 20 ms. Dynamic exclusion time was set as 40 sec and the cycle time of each DDA cycle is set as 3 sec. The MS2 Orbitrap resolution was set at 7,500 with standard AGC target and the maximum injection time were set at $1 \times 10^5$ and 30 ms, respectively.

*Annexin-V apoptosis assay*. Cells were stained by FITC-conjugated Annexin-V (BioVision) and propidium iodide (PI) (Invitrogen) in Annexin-V binding buffer (BD Biosciences) at room temperature for 30 minutes. Apoptosis percentage was determined using BD Accuri C6 flow cytometer and FACSDiva software (BD Biosciences).

*STRING analysis.* Protein candidates with unique peptide greater than 2 were input into the STRING platform (https://string-db.org/) for protein-protein interaction analysis [4].

*Molecular Docking*. The molecular interactions between FABP4 and ITGB1 was investigated by conducting a comprehensive protein–protein docking analysis. High-resolution crystal structures of FABP4 (PDB ID: 7FWG) and ITGB1 (PDB ID: 3T9K) were retrieved from the RCSB Protein Data Bank [5] (accessed on 2$^{nd}$ June, 2025). All non-protein entities, including ligands and water molecules, were meticulously removed using BIOVIA Studio Visualizer 2021 to ensure the structural integrity for docking. The processed PDB files were submitted to the LZerD Protein Docking Webserver [6,7], utilizing default parameter settings for clustering cutoff (4.0) and surface coarsen max cost (0.0001). The resulting docked complex was subsequently visualized in PyMOL [8], enabling detailed three-dimensional structural interrogation and high-quality image generation.

*Western blot analysis.* Whole cell lysates were extracted using either NETN buffer supplemented with protease inhibitor cocktail or direct lysis. Protein lysate was separated by SDS-polyacrylamide gel electrophoresis (SDS-PAGE) and transferred to polyvinylidene difluoride membrane (Millipore) for western blot analyses. Primary antibodies FABP4 [EPR3579] (1:1000, ab92501, Abcam), pAKT(Ser473) (D9E), pGSK3β (Ser9) (D3A4), AKT and β-

catenin (D10A8) (1:1000,#4060S, #9322S, #9272S & #8480S, Cell Signaling Technology), GSK3β (clone 7) (1:1000, #610202, clone 7, BD Transduction Laboratories), DDK (OTI4C5) (1:1000,TA50011-100, Origene), ITGB1 (1:1000, 12594-1-AP, Proteintech) and β-ACTIN (AC-74) (1:5000, #A5316, Sigma Aldrich) were incubated at 4°C overnight. After washing, the membrane was incubated with horseradish peroxidase-conjugated anti-mouse or rabbit antibody (GE HealthCare). The signals were visualized using the enhanced chemiluminescence method. Blot images were quantified by densitometry using ImageJ software. The protein expression was normalized to β-actin and expressed as fold change relative to respective controls.

*Immunohistochemical analysis of resected mouse tumors.* Sections were deparaffinized in xylene and rehydrated in graded alcohols and distilled water. Slides were processed for antigen retrieval by a standard microwave heating technique in Tris-EDTA buffer. Endogenous peroxidase activities were quenched using 3% hydrogen peroxide. The sections were immersed in serum-free-protein block solution (DAKO). Specimens were subsequently incubated with primary antibodies (PCNA (PC10): 1:5000, ab29, Abcam; α-SMA: 1:500, ab5694, Abcam; β-catenin (D10A8): 1:100, #8480S, Cell Signaling Technology). The sections were then washed thoroughly and incubated with anti-rabbit Envision™ HRP-conjugated secondary antibody (DAKO). Positive signals were visualized using Liquid DAB+ Substrate-Chromogen System (DAKO). Sections were counterstained with Mayer's hematoxylin followed by examination using light microscope. The number of positive PCNA nuclei and expression of β-catenin in mouse tumor tissues were quantified using ImageJ software.

*Multiplexed fluorescent immunohistochemistry in human fatty liver tissue and resected mouse liver tumors.* The tyramide signal amplification-based method was used for staining multiple targets in HCC paraffin embedding specimens with Opal 4-Color Manual IHC Kit (NEL810001KT, Akoya Biosciences). Sections were deparaffinized in xylene and rehydrated in decreasing graded alcohols and distilled water. Slides were processed for antigen retrieval by a standard inverter microwave heating technique with either diluted 50x Envision FLEX Target Retrieval Buffer (pH9.0, K8004, Dako) for FABP4, pGSK3β (Ser9), pAKT (Ser473), CD68, CD86, CD206 and CD31 or 10x AR6 sodium citrate buffer for Perilipin-1 and β-catenin (pH6.0, AR600250ML, Akoya BioSciences) for 15 minutes. Endogenous peroxidase activities were quenched using 3% hydrogen peroxide for 10 minutes at room temperature. The sections were immersed in blocking/antibody diluent (ARD1001EA, Akoya Biosciences) for 30 minutes at room temperature. Specimens were incubated with primary antibodies overnight at 4℃ (FABP4 [EPR3579] and Perilipin-1 (1:1000 for ab92501 and 1:200 for ab3526, Abcam), pGSK3β (Ser9) (D3A4), pAKT (Ser473) (D7F10), F4/80 (D2S9R), CD68 (E3O7V), CD86 (E5W6H) and CD206 (E6T5J) (1:500 for #9322S, 1:200 for #9018S, 1:200 for #70076T, 1:500 for #97778S, 1:500 for #19589S and 1:500 for #24595S, Cell Signaling Technology). For β-catenin (D10A8, #8480S, Cell Signaling Technology) and CD31 (F2N3M, #15585T, Cell Signaling Technology), primary antibodies were incubated at 1:100 or 1:500 for 3 hours at room temperature. The sections were then washed thoroughly and incubated with Opal polymer HRP Mouse+Rabbit (ARH1001EA, Akoya Biosciences) for 30 minutes at room temperature. Followed by a brief wash with 1xTBST, Opal fluorophore (1:100) was applied for FABP4, pGSK3β (Ser9) or CD86 (Opal 520), Perilipin-1, pAKT (Ser473), F4/80 or CD68 (Opal 570) and β-catenin, CD206 or CD31 (Opal 690) for 15 minutes at room temperature. A final stripping step was performed with diluted 10x AR6 sodium citrate buffer in the microwave

oven for 15 minutes. The section slides were cooled down, counterstained with DAPI solution (1:1000) and mounted for examination using Leica TCS SPE confocal microscope. The fluorescent intensity was quantified using ImageJ software. Colocalization analysis was conducted using Coloc2 plugin of ImageJ software. Pearson's correlation coefficient was used to express the correlation between variables of interest [9].

β-catenin TCF binding luciferase reporter assay. β-catenin activity was examined using luciferase reporter assay of TCF/LEF-dependent transcription (TOP/FOPFLASH reporter assay). Either firefly luciferase pSuper8XTOPflash or pSuper8XFOPflash constructs (gifts from Dr. Moon R, University of Washington), together with Renilla luciferase construct pRL-CMV (Promega) for normalization of transfection efficiency, were transfected using Lipofectamine® 2000. Luciferase activities were assayed using Dual-Luciferase® Reporter Assay System (Promega) according to manufacturer’s protocol.

*Immunoprecipitation.* The protein lysate was incubated with anti-DDK antibody (OTI4C5) (1:1000, TA50011-100, Origene), together with Protein A agarose beads (#9863, Cell Signaling Technology) at 4°C overnight with gentle agitation. Normal mouse IgG served as control. The immunoprecipitates were eluted by boiling in 2X SDS loading buffer for 10 minutes and then subjected to SDS-PAGE and immunoblotting analyses.

*In vivo tumorigencity assay.* In vivo evaluation of tumorigenicity was performed with either male nonobese diabetic/severe combined immunodeficient (NOD/SCID) or NOD/SCID gamma mice of age 4-6 week-old by induction of tumor xenografts. Cells were suspended in 1:1 culture medium and BD Matrigel Matrix (BD Biosciences) and subcutaneously injected into the flanks

of the mice, which were kept under observation. Briefly, each mouse received two injections of cells in both flanks, and cells pretreated with either rhFABP4 (100 ng/mL) or ADCM for 24 hours with their control group were injected into NOD/SCID and NOD/SCID gamma mice, respectively. Tumors were harvested at the end of the experiment for documentation. Tumor-initiating cell frequency was calculated using Extreme Limiting Dilution Analysis (ELDA) software [3]. No specific randomization method was used. Sample size of animals was chosen based on significant *p* values. The study protocol was approved by and performed in accordance with the guidelines for the Use of Live Animals in Teaching and Research at Hong Kong Polytechnic University. The study protocol was approved by and performed in accordance with the guidelines for the Use of Live Animals in Teaching and Research at Hong Kong Polytechnic University. The tumor volumes did not exceed 10% of normal body weight or 1.3 cm in diameter. Mice were sacrificed if the percentage of body weight loss is greater than 20% including the tumor mass. The maximal tumor size/burden was permitted by study protocol of the Hong Kong Polytechnic University. The maximal tumor size/burden was not exceeded in all experiments.

*RNA extraction and quantitative PCR (qRT-PCR) analysis.* Total RNA was isolated using TRIzol reagent according to the manufacturer's protocol (Invitrogen). Complementary DNA (cDNA) was synthesized using 1μg total RNA with PrimeScript RT Reagent Kit (Takara Bio, Shiga, Japan) according to the manufacturer's instructions and then subjected to qPCR with BrightGreen 2x qPCR Master mix (Applied Biological Materials Inc) using QuantStudio 7 Flex Read Time PCR System (Applied Biosystems, Foster City, California, US) with primers specific to the sequences of genes of interest which were provided in **Supplemental Table 5**. Relative

expression differences were calculated using $2^{-\Delta\Delta CT}$method with reference to either *GAPDH* or *Actb*.

*RNA sequencing*. Total RNA of rhFABP4 0 ng/mL (control) or 100 ng/mL treated PLC/PRF/5 and resected tumors from WT and *Fabp4*$^{-/-}$ mouse in chemical induced HFD model were extracted using TRIzol Reagent (Life Technologies) according to manufacturer's protocol. The quality of total RNA was checked by Agilent 2100 bioanalyzer (Agilent Technologies Inc.) to have OD260/280 ratio of between 1.8-2.0 and RNA integrity number (RIN) of value higher than 8.0. The RNA samples which met the quality assessment were then subjected to Illumina sequencing using Hiseq 1500 sequencer (Illumina) or NextSeq2000 (Illumina) for performing sequencing run. Each pair-end sample had an average throughput of 20Gb.

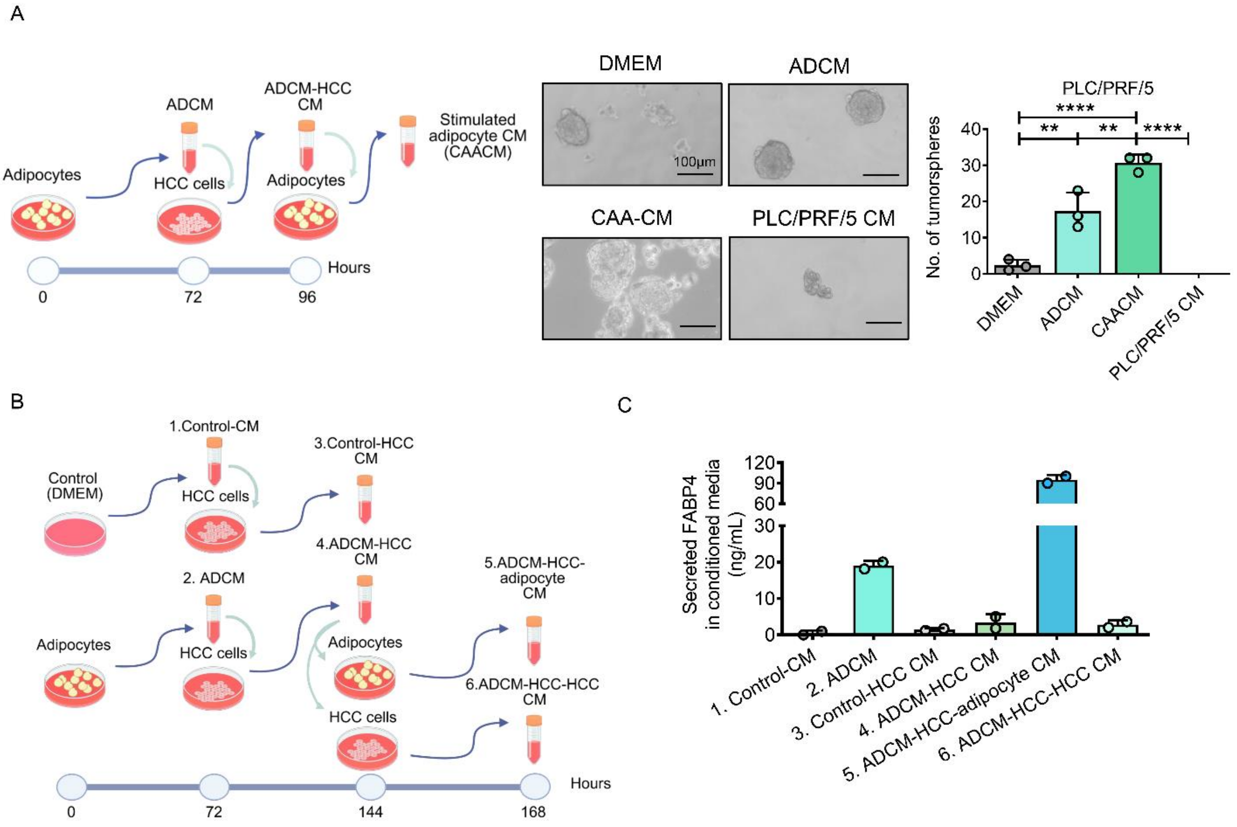


**Supplemental Figure 1. The secretion of FABP4 in adipocytes was stimulated by conditioned medium from adipocyte conditioned medium (ADCM)-incubated HCC cells.** (**A**) Schematic diagram showing the workflow of stimulated adipocyte CM (CAACM) collection. The tumorsphere formation of PLC/PRF/5 was enhanced by ADCM when compared to DMEM control, which was further elevated by CAACM ($n$=3, **$p$<0.01 & ****$p$<0.0001, one-way ANOVA followed by Tukey's multiple comparisons test). Scale bar = 100 µm. (**B**) Schematic diagram showing the workflow of CM collection from stimulated adipocytes or HCC cells. (**C**) Secreted FABP4 levels in conditioned media were detected using ELISA ($n$=2). Data was presented as the mean ± SD.

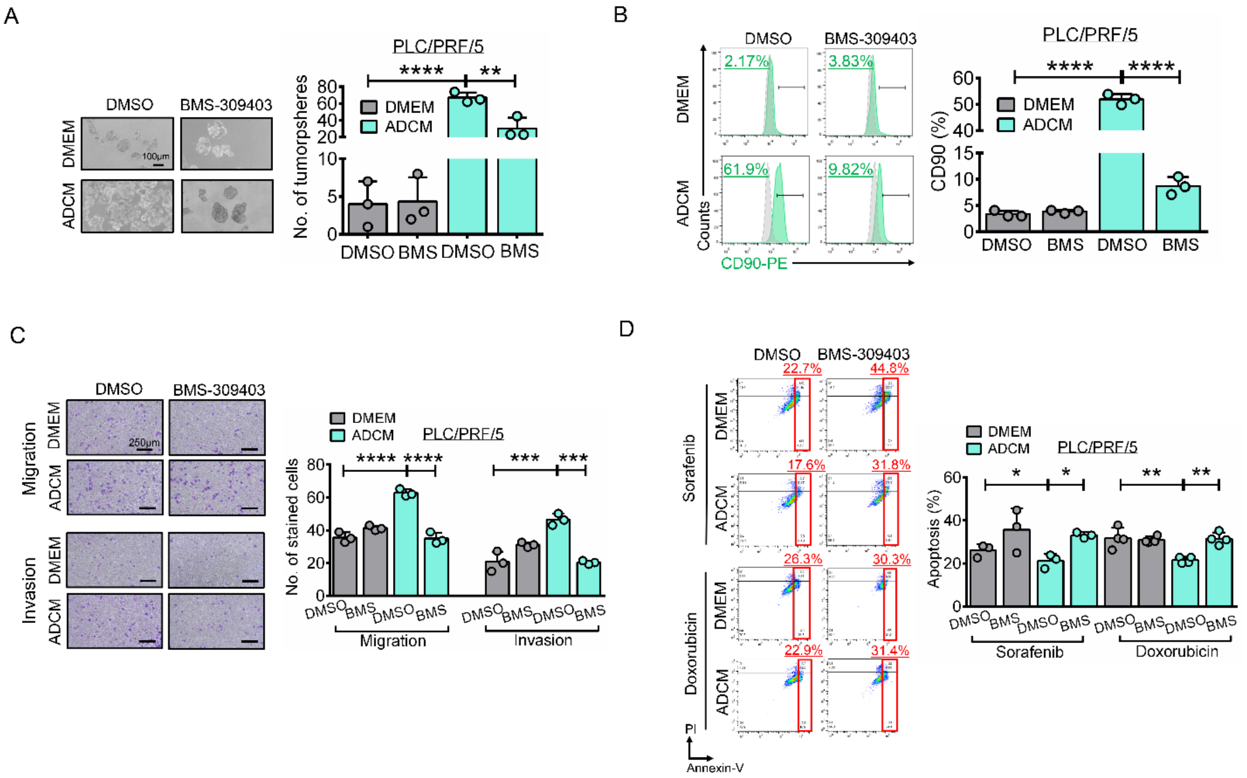


**Supplemental Figure 2. Inhibition of FABP4 in ADCM by BMS-309403 attenuated the CSC properties of HCC cells.** (**A**) In vitro tumorsphere formation assay demonstrated BMS-309403 at 1 µM suppressed the enhancing self-renewal ability of ADCM conferred to PLC/PRF/5 (*n*=3). Representative images showing the tumorspheres. Scale bar: 100 µm. (**B**) Expression of liver CSC marker CD90 was measured by flow cytometry analysis. (*n*=3). (**C**) The migration and invasive abilities of HCC cells was evaluated by uncoated (top) and Matrigel-coated transwell (bottom) assays, respectively (*n*=3). Representative images of stained cells were shown. Scale bar: 250 µm. (**D**) The apoptosis of cotreatment of ADCM with BMS-309403 HCC cells induced by doxorubicin (1 µg/mL) or sorafenib (6 µM) was measured using Annexin-V PI staining (Doxorubicin treatment: *n*=4 while sorafenib treatment: *n*=3). Data was presented as the mean ± SD. **p*<0.05, ***p*<0.01, ****p*<0.001 & *****p*<0.0001, one-way ANOVA followed by Tukey's multiple comparisons test.

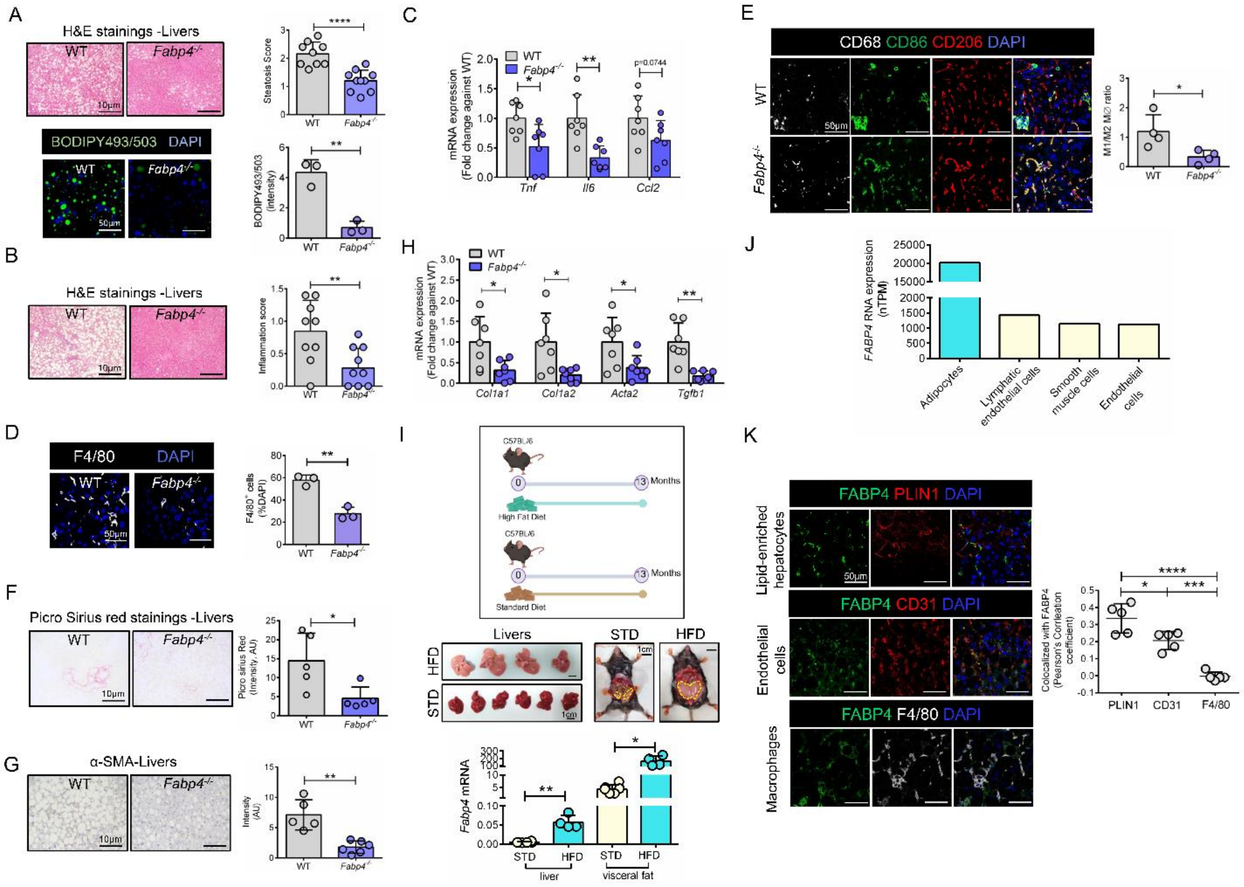

**Supplemental Figure 3. Genetic loss of *Fabp4* dampened steatosis and inflammation of the livers upon DEN-HFD.** (**A**) Steatosis of mouse livers from WT and *Fabp4*$^{-/-}$ was assessed using H&E (WT *n*=9 & *Fabp4*$^{-/-}$ *n*=10 mice) and BODIPY™493/503 staining (*n*=3 mice). Representative H&E and BODIPY™493/503 images were shown. The tissues were counterstained with either haematoxylin or DAPI (blue). Scale bar: 10 µm and 50 µm. (**B**) Lobular inflammation score was assessed. Scale bar: 10 µm. (**C**) Expression of pro-inflammatory genes of livers were measured (*n*=7 mice). (**D**) The level of F4/80 (white) macrophages was examined in the livers (*n*=3 mice). The tissues were counterstained with DAPI (blue). Scale bar: 50 µm. (**E**) Multiplex IHC staining on M1 and M2 macrophages (MØ) were performed using combination of CD68 (white)/CD86 (green) or CD68 (white)/CD206 (red) (*n* = 4 mice). Scale bar: 50 µm. Number of positive cells was quantified and expressed as M1/M2 MØ ratio. (**F-G**) Picro sirius red and α-SMA stainings of mouse livers from WT and *Fabp4*$^{-/-}$ (*n*=5-6 mice). Scale bar: 10 µm. (**H**) Expression of fibrosis-related genes of livers were measured (*n*=7 mice). (**I**) Schematic diagram showing the long-term feeding of high-fat diet (HFD) in mice. *Fabp4* level of livers and visceral fat was examined (STD *n*=6 & HFD *n*=4 mice). Representative images of livers and visceral fat (yellow dotted circle) were presented. Scale bar: 1 cm. (**J**) RNA expression of human *FABP4* in different cell types from The Human Protein Altas (cutoff of nTPM>10000). (**K**) Colocalization analyses of FABP4 (green), PLIN1 (red), CD31 (red), F4/80 (white) and counterstained with DAPI (blue) (*n*=5 mice). **p*<0.05,***p*<0.01, ****p*<0.001 & ****p<0.0001. A-I: two-tailed *t* test; K: one-way ANOVA followed by Tukey's multiple comparisions test. Data was presented as mean ± SD.

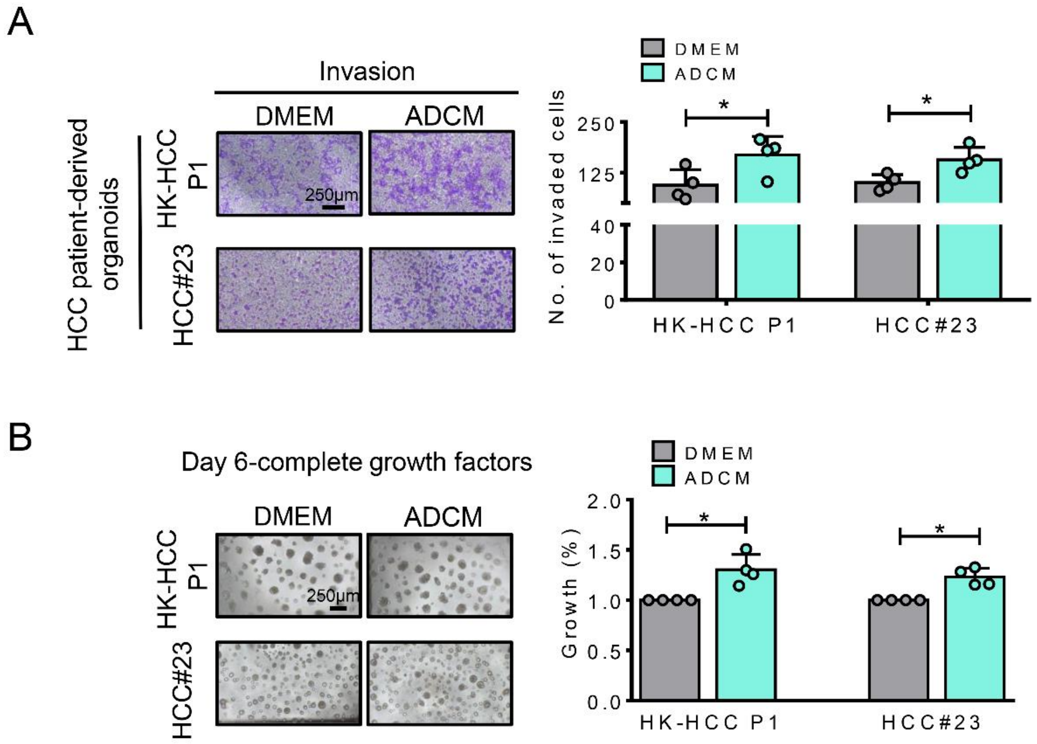


**Supplemental Figure 4. Effect of adipocyte-conditioned medium (ADCM) on HCC patient-derived organoids.** (**A**) ADCM enhanced cell invasiveness of HCC patient-derived organoids in matrigel-coated transwell assay (*n*=4). Scale bar: 250 µm. (**B**) Representative images of organoids upon treatment of ADCM were shown. The growth of HCC patient-derived organoids was significantly enhanced upon administration of ADCM for 6 days using Cell-Titer-Glo assay (*n*=4). Scale bar: 250 µm. Data was presented as the mean ± SD. **p*<0.05, two-tailed *t* test.

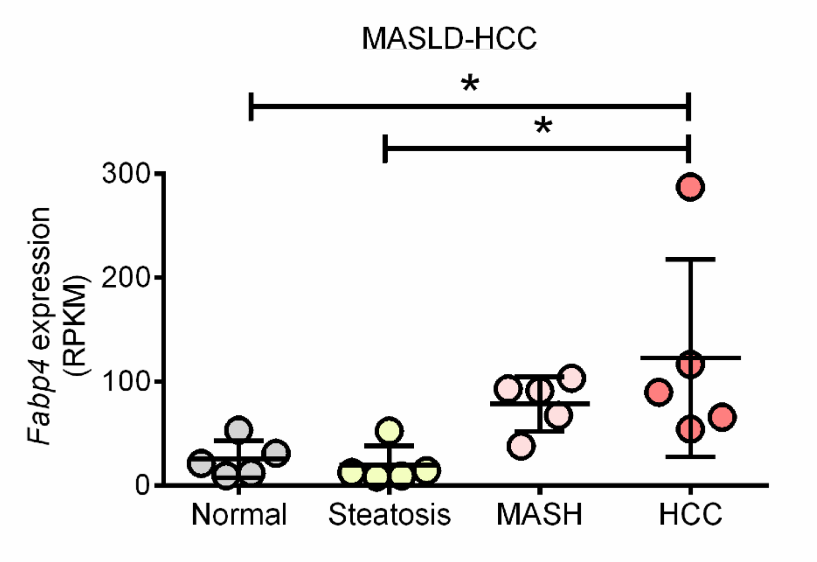


**Supplemental Figure 5. *Fabp4* mRNA expression in various stages of MASLD-HCC mouse model.** Bulk RNA sequencing of MASLD-HCC mouse model (*n*=5 mice). Data was presented as the mean ± SD. *$p$<0.05, one-way ANOVA followed by Tukey's multiple comparisons test.

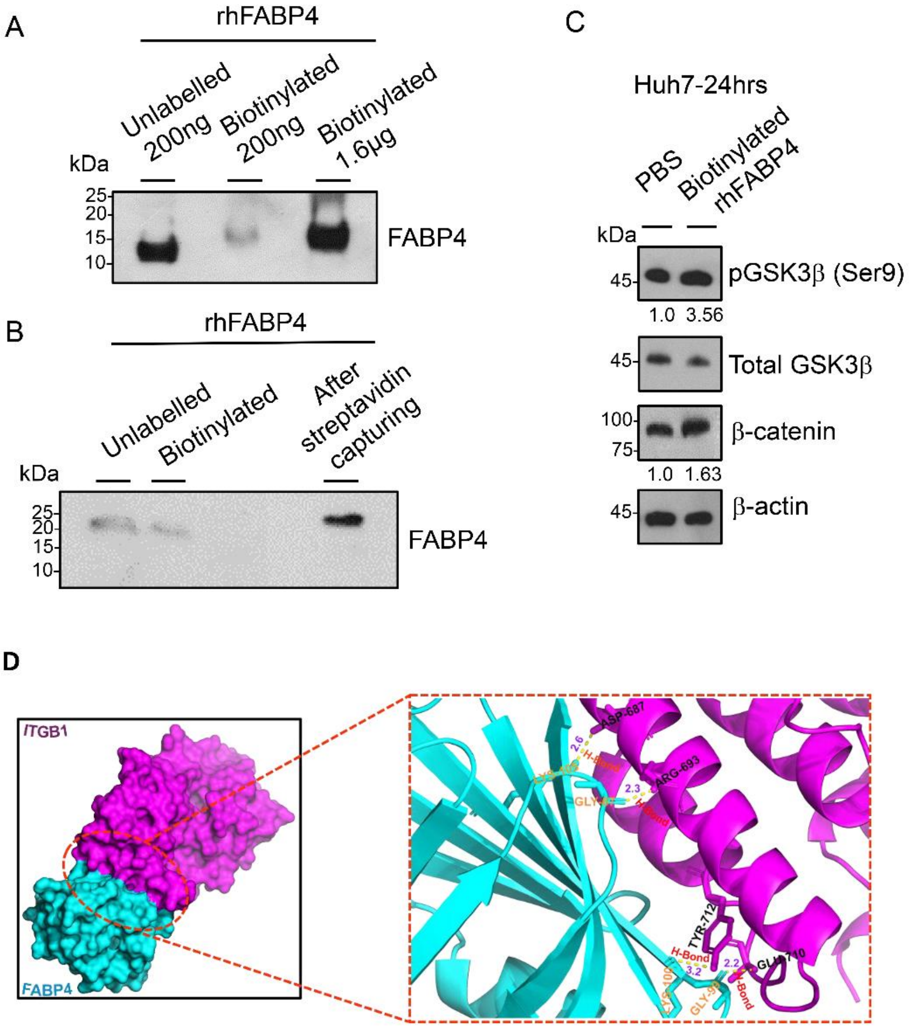


**Supplemental Figure 6. Biotinylation of recombinant FABP4 protein (rhFABP4) for cell surface target binding identification using mass spectrometry and molecular docking analysis of FABP4-ITGB1 complex.** (**A**) Western blot analysis showing the successful detection of FABP4 protein after biotinylation. (**B**) Biotinylated FABP4 was detected after capturing of streptavidin. (**C**) The function of rhFABP4 was not affected upon biotinylation, as demonstrated by the upregulation of pGSK3β (Ser9) and β-catenin in Western blot analyses. (**D**) Structural model of FABP4-ITGB1 complex. Protein-protein interface and key interfacial hydrogen bonds were highlighted. FABP4 (cyan) and ITGB1 (magenta) interacted via a defined network of residues (LYS-105 and GLY-67 of FABP4 formed strong hydrogen bonds with ASP-687 and ARG-693 of ITGB1 at 2.6 Å and 2.3 Å, respectively, whereas LYS-100 and GLY-99 of FABP4 interacted with TYR-712 and GLU-710 of ITGB1 at 3.2 Å and 2.2 Å, respectively).

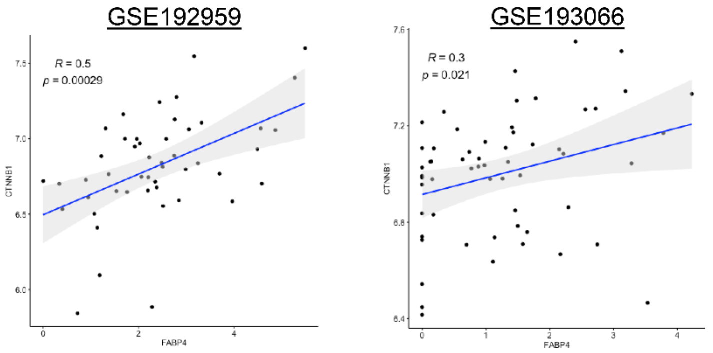


(N=48, liver biopsy collected ablating MASLD-related HCC tumor)

(N=58, MASLD patients with high HCC risk)

**Supplemental Figure 7. *FABP4* expression was positively correlated with *CTNNB1* in MASLD-related HCC patients and MASLD patients at high risk of HCC.** According to the GSE192959 and GSE193066 datasets, *FABP4* was positively correlated with *CTNNB1* in MASLD-related HCC patients and MASLD patients at high risk of HCC (R=0.5, *p*=0.00029 & R=0.3, *p*=0.021; Pearson correlation).

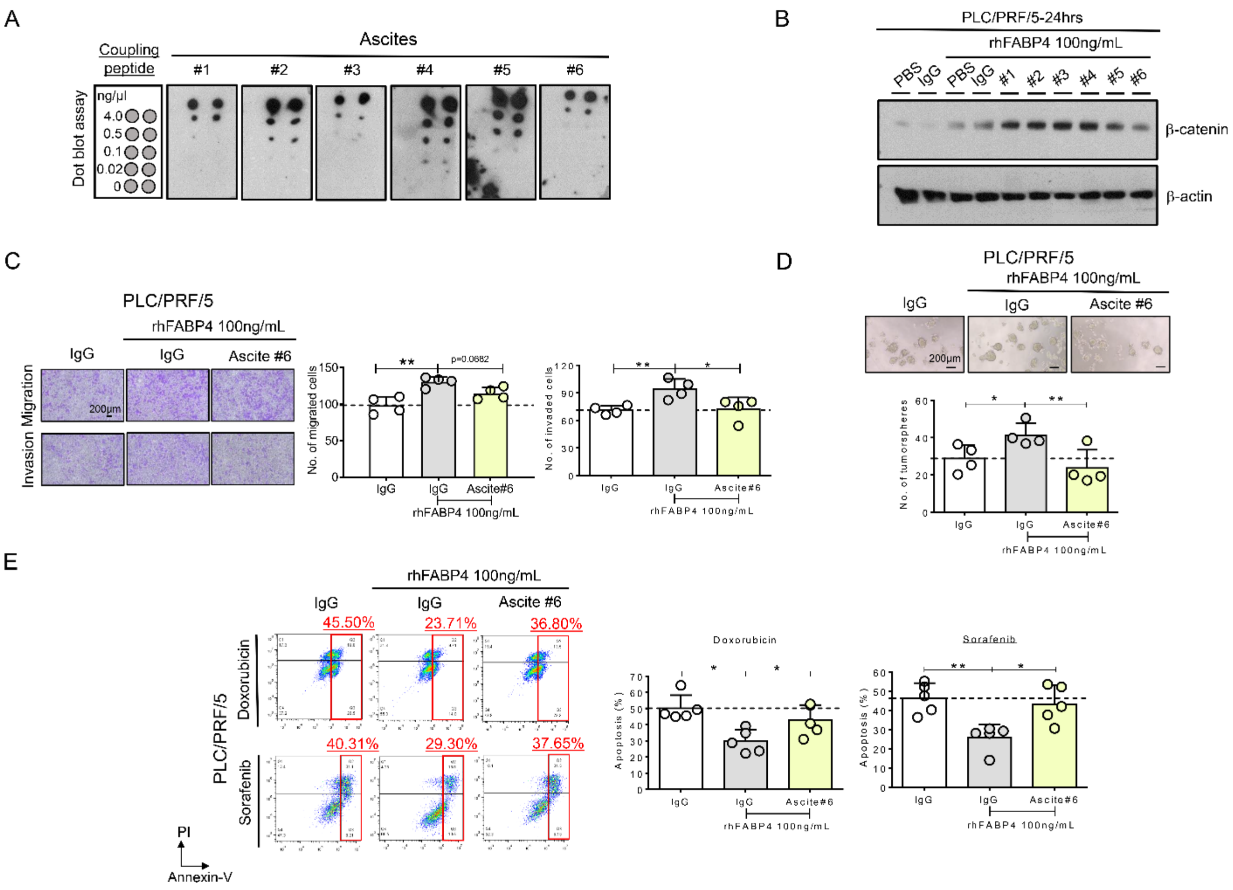


**Supplemental Figure 8. Generation of anti-human FABP4 monoclonal antibody based on the functional characterization of the mouse ascites.** (**A**) The detection efficiency of the six ascites was examined using coupling peptide in dot blot assay. Representative dot blot results were shown. (**B**) Cotreatment of rhFABP4 with ascites #1-#6 at 10 µg/mL for 24 hours were performed using PLC/PRF/5. Western blot analyses showed that ascite #6 repressed the upregulation of β-catenin. Ascite #6 was chosen for functional characterization. (**C**) The effects of rhFABP4 at 100 ng/mL in cell migratory and invasive abilities were abolished by treatment of ascite #6 at 2 µg/mL when compared with normal IgG control (*n*=4). Scale bar: 200 µm. (**D**) The enhanced self-renewing ability by rhFABP4 was abrogated by cotreatment with ascite#6 at 1 µg/mL (*n*=4). Scale bar: 200 µm. (**E**) Drug resistance conferred by rhFABP4 were rescued by ascite #6 upon treatment of either doxorubicin (1.5 µg/mL) or sorafenib (6 µM) (*n*=5). Data was presented as the mean ± SD. *$p<0.05$ & **$p<0.01$, one-way ANOVA followed by Tukey's multiple comparisons test.

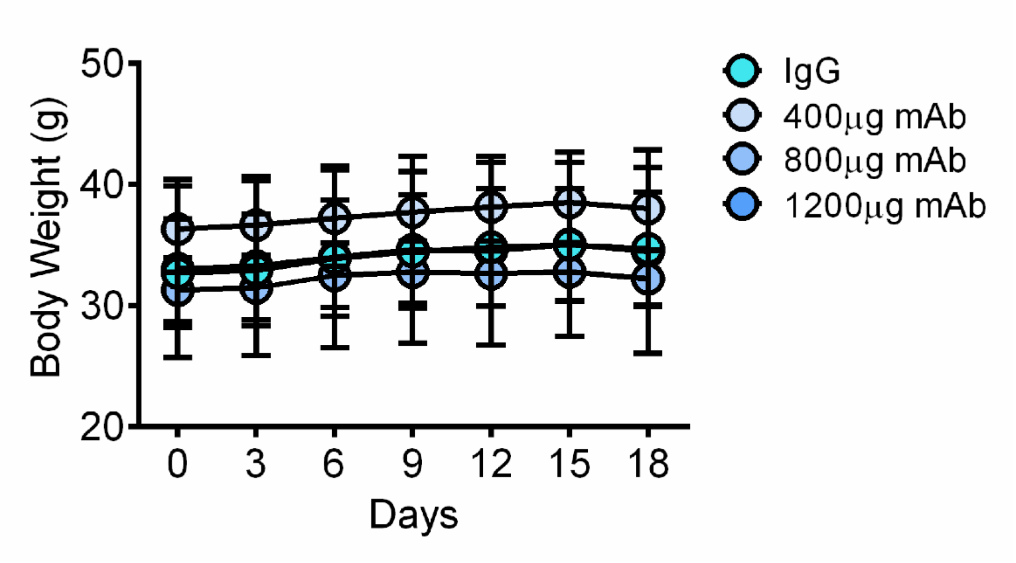


**Supplemental Figure 9. Effect of anti-FABP4 monoclonal antibody on body weight of the mice.** No notable change in body weight was observed after treatment of anti-FABP4 mAb ranging from 400 μg to 1200 μg (*n*=6 mice per group). Data was presented as the mean ± SD.

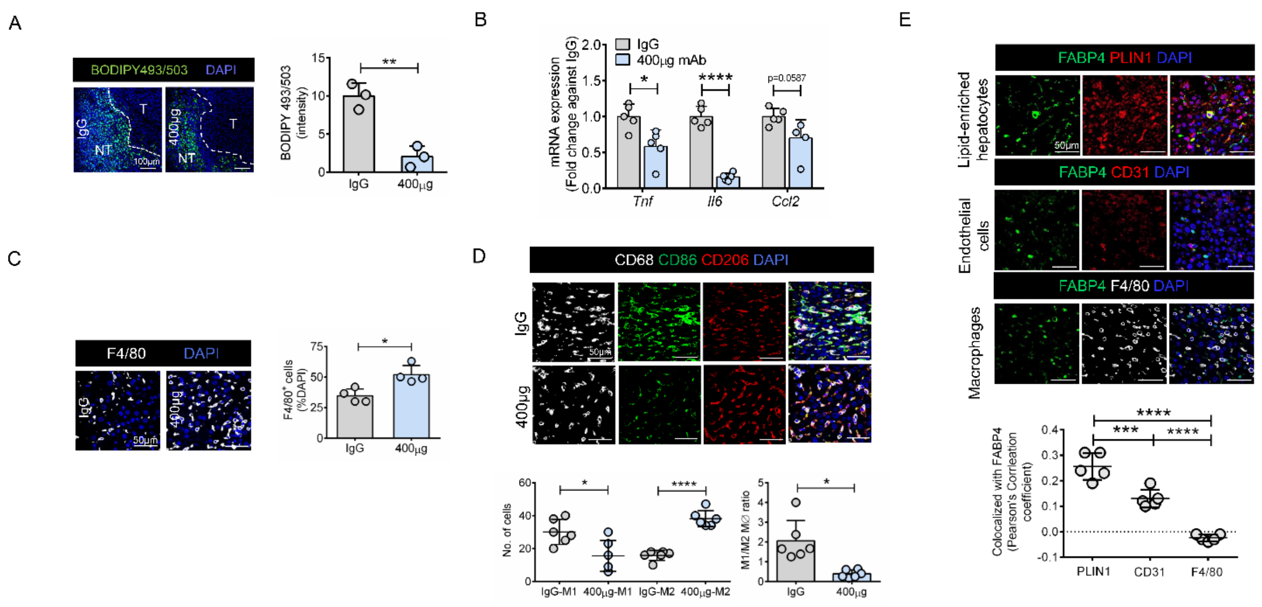


**Supplemental Figure 10. Neutralization of FABP4 by monoclonal antibody (3I19-1) approach in MASLD-HCC mouse model suppressed the tumor growth through modulating of immune environment in the liver.** (**A**) The accumulation of neutral lipid droplets in the livers was reduced upon administration of anti-FABP4 monoclonal antibody at 400 µg (*n*= 3 mice). Representative BODIPY$^{TM}$493/503 images were shown (NT: non-tumor, T: tumor). The tissues were counterstained with DAPI (blue). Intensity of BODIPY$^{TM}$493/503 was quantified using ImageJ. Scale bar: 100 µm. (**B**) Expression of pro-inflammatory genes *Tnf*, *Il6* and *Ccl2* in mouse livers was examined (*n*=5 mice). (**C**) The level of F4/80 macrophages was examined in the livers (*n*=4 mice). The tissues were counterstained with DAPI (blue). Positive staining was quantified using ImageJ. Scale bar: 50 µm. (**D**) Multiplex IHC staining on M1 and M2 macrophages (MØ) were performed using combination of CD68 (white)/CD86 (green) or CD68 (white)/CD206 (red) (*n* = 6 mice). Scale bar = 50 µm. The tissues were counterstained with DAPI (blue). Number of positive cells was quantified using ImageJ and expressed as M1/M2 MØ ratio. (**E**) Colocalization analyses of FABP4 (green), PLIN1 (red), CD31 (red), F4/80 (white) and counterstained with DAPI (blue) (*n*=5 mice). Data was presented as mean ± SD. *$p$<0.05, **$p$<0.01, ***$p$<0.001 & ****$p$<0.0001. A-D: two-tailed $t$ test; E: one-way ANOVA followed by Tukey's multiple comparisons test.

**Supplemental tables**

**Supplemental Table 1**. Effect of adipocyte conditioned medium (ADCM) on tumorigenicity of PLC/PRF/5 and Huh7 cells. (A) Subcutaneous in vivo tumor development in NOD/SCID gamma (NSG) mice of pretreated PLC/PRF/5 cells with either DMEM (control) or ADCM. (B) Subcutaneous in vivo tumor development in NSG mice of pretreated Huh7 cells with either DMEM or ADCM. Significance was calculated by extreme limiting dilution analysis with chi-square test.

(A) Primary engraftment of PLC/PRF/5 cells

| | **Tumor incidence rate** | | | **Extreme limiting dilution** | | |
|---|---|---|---|---|---|---|
| | $5X10^3$ cells | $1x10^4$ cells | $5x10^4$ cells | Estimated CSC frequency | 95% CI | *p*-value |
| **DMEM** | 3/9 | 3/9 | 4/5 | 1/22710 | 1/11328-1/45528 | 3.26e-06 |
| **ADCM** | 9/9 | 7/8 | 5/5 | 1/2685 | 1/5262-1/1370 | |

(B) Primary engraftment of Huh7 cells

| | **Tumor incidence rate** | | | **Extreme limiting dilution** | | |
|---|---|---|---|---|---|---|
| | $5X10^3$ cells | $1x10^4$ cells | $5x10^4$ cells | Estimated CSC frequency | 95% CI | *p*-value |
| **DMEM** | 1/5 | 2/5 | 3/5 | 1/36748 | 1/88192-1/15312 | 0.00902 |
| **ADCM** | 3/5 | 3/5 | 5/5 | 1/8033 | 1/18175-1/3551 | |

**Supplemental Table 2.** Key adipocyte-specific secretory proteins in ADCM determined by mass spectrometry. A list of top 22 adipocyte-specific secretory proteins that were identified in ADCM was shown. Score of zero is given to protein that was not detected in CAACM or HCCCM.

| | Protein | Protein score (ADCM) | Protein score (CAACM) | Fold Change (CAACM-ADCM)/ADCM) | Fold Change (HCCCM-ADCM)/ADCM |
|---|---|---|---|---|---|
| 1 | Laminin subunit gamma-1 (LAMC1) | 913 | 810 | -0.11 | -0.55 |
| 2 | Gelsolin (GSN) | 547 | 609 | -0.11 | -0.64 |
| 3 | Pentraxin-related protein PTX3 (PTX3) | 494 | 231 | -0.53 | -1 |
| 4 | Laminin subunit alpha-4 (LAMA4) | 380 | 139 | -0.63 | -1 |
| 5 | Tenascin C (TNC) | 353 | 23 | -0.93 | -1 |
| 6 | Lumican (LUM) | 258 | 266 | 0.031 | -0.90 |
| 7 | Annexin A5 (ANXA5) | 253 | 353 | 0.39 | -1 |
| 8 | Nidogen-1 (NID1) | 221 | 215 | -0.02 | -1 |
| 9 | Calmodulin (CALM) | 211 | 1218 | 4.77 | 1.72 |
| 10 | Legumain (LGMN) | 163 | 115 | -0.29 | -0.63 |
| 11 | Alpha-2-HS-glycoprotein (AHSG) | 126 | 214 | 0.69 | 1.31 |
| 12 | Periostin (POSTN) | 114 | 0 | -1 | -1 |
| 13 | Thymosin beta-10 (TMSB10) | 95 | 98 | 0.03 | 2.66 |
| 14 | Phospholipid transfer protein (PLTP) | 65 | 0 | -1 | -1 |
| 15 | Adipocyte enhancer-binding protein 1 (AEBP1) | 65 | 0 | -1 | -1 |
| 16 | Macrophage colony-stimulating factor 1 (CSF1) | 59 | 69 | 0.17 | -1 |
| 17 | Serglycin (SRGN) | 59 | 0 | -1 | -1 |
| 18 | Golgi membrane protein 1 (GOLM1) | 50 | 523 | 9.46 | 7.58 |
| 19 | Olfactomedin-like protein 3 (OLFL3) | 50 | 0 | -1 | -1 |
| 20 | Dickkopf-related protein 3 (DKK3) | 40 | 0 | -1 | -1 |
| 21 | Fatty acid-binding protein (FABP4) | 33 | 324 | 8.8 | -1 |
| 22 | Xaa-Pro dipeptidase (PEPD) | 27 | 76 | 1.8 | -1 |

**Supplemental Table 3.** Effect of recombinant human FABP4 (rhFABP4) on tumorigenicity of PLC/PRF/5 and Huh7 cells. (A) Subcutaneous in vivo tumor development in NOD/SCID mice of pretreated PLC/PRF/5 cells with either PBS (control) or rhFABP4 at 100 ng/mL. (B) Subcutaneous in vivo tumor development in NOD/SCID mice of pretreated Huh7 cells with either PBS or rhFABP4 at 100 ng/mL. Significance was calculated by extreme limiting dilution analysis with chi-square test.

(A) Primary engraftment of PLC/PRF/5 cells

| | **Tumor incidence rate** | | **Extreme limiting dilution** | | |
|---|---|---|---|---|---|
| | $1X10^3$cells | $5x10^3$cells | Estimated CSC frequency | 95% CI | *p*-value |
| **PBS** | 2/5 | 1/5 | 1/8749 | 1/29109-1/2630 | 0.0373 |
| **rhFABP4** | 3/5 | 4/5 | 1/2108 | 1/5182-1/858 | |

(B) Primary engraftment of Huh7 cells

| | **Tumor incidence rate** | | | **Extreme limiting dilution** | | |
|---|---|---|---|---|---|---|
| | $1X10^3$ cells | $5x10^3$ cells | $1x10^4$ cells | Estimated CSC frequency | 95% CI | *p*-value |
| **PBS** | 1/5 | 2/5 | 1/5 | 1/17193 | 1/47765-1/6189 | 0.00435 |
| **rhFABP4** | 3/5 | 4/5 | 4/5 | 1/3404 | 1/7177-1/1615 | |

**Supplemental Table 4. shRNA and sgRNA sequences.**

| | **Sequence (5'-3')** |
|---|---|
| **NTC** | CCGGTTGTGCTCTTCATCTTGTTGCCGGCAACAAGATGAAGAGCACCAATTTTTG |
| **sh*ITGB1*#1** | GCCTCCAGATGACATAGAAA |
| **sh*ITGB1*#2** | GCCTTGCATTACTGCTGATAT |
| **sh*CTNNB1*#1** | GCGCATGGAAGAAATAGTTGAA |
| **sh*CTNNB1*#2** | GCCTTTAGCTGTATTGTCTGAA |

**Supplemental Table 5. Primer sequences for qRT-PCR.**

| Genes | Forward primer (5'-3') | Reverse primer (5'-3') |
|---|---|---|
| ***FABP4*** | ACTGGGCCAGGAATTTGACG | ACGCATTCCACCACCAGTTTA |
| ***PPARG*** | GCAAACCCCTATTCCATGCTG | GTGTCAACCATGGTCATTTCTTGT |
| ***GAPDH*** | CCGGGAAACTGTGGCGTGATGG | AGGTGGAGGAGTGGGTGTCGCTGTT |
| ***Ccl2*** | TAAAAACCTGGATCGGAACCAAA | GCATTAGCTTCAGATTTACGGGT |
| ***Il6*** | CTGCAAGAGACTTCCATCCAG | AGTGGTATAGACAGGTCTGTTGG |
| ***Tnf*** | GGTGCCTATGTCTCAGCCTCTT | GCCATAGAACTGATGAGAGGGAG |
| ***Fabp4*** | AAGGTGAAGAGCATCATAACCCT | TCACGCCTTTCATAACACATTCC |
| ***Actb*** | GGCTGTATTCCCCTCCATCG | CCAGTTGGTAACAATGCCATGT |
| ***Col1a1*** | GCTCCTCTTAGGGGCCACT | CCACGTCTCACCATTGGGG |
| ***Col1a2*** | GGAACAAATGGGCTCACTGG | GCTCACCCTTGTTACCGGAT |
| ***Acta2*** | GTCCCAGACATCAGGGAGTAA | TCGGATACTTCAGCGTCAGGA |
| ***Tnfb1*** | GTCACTGGAGTTGTACGGCA | GAAGGGCCGGTTCATGTCAT |

**Supplemental references**


1. Kleiner DE, et al. Design and validation of a histological scoring system for nonalcoholic fatty liver disease. *Hepatology*. Jun 2005;41(6):1313-21.

2. Huch M, et al. Long-term culture of genome-stable bipotent stem cells from adult human liver. *Cell*. Jan 15 2015;160(1-2):299-312.

3. Hu Y, et al. ELDA: extreme limiting dilution analysis for comparing depleted and enriched populations in stem cell and other assays. *J Immunol Methods*. Aug 15 2009;347(1-2):70-8.

4. Szklarczyk D, et al. The STRING database in 2023: protein-protein association networks and functional enrichment analyses for any sequenced genome of interest. *Nucleic Acids Res*. Jan 6 2023;51(D1):D638-D646.

5. Berman HM, et al. The Protein Data Bank. *Nucleic Acids Res*. Jan 1 2000;28(1):235-42.

6. Christoffer C, et al. LZerD webserver for pairwise and multiple protein-protein docking. *Nucleic Acids Res*. Jul 2 2021;49(W1):W359-W365.

7. Christoffer C, et al. LZerD Protein-Protein Docking Webserver Enhanced With de novo Structure Prediction. *Front Mol Biosci*. 2021;8:724947.

8. The PyMOL Molecular Graphics System, Version 3.0 Schrödinger, LLC.

9. Dunn KW, et al. A practical guide to evaluating colocalization in biological microscopy. *Am J Physiol Cell Physiol*. Apr 2011;300(4):C723-42.
