# Supplementary material for: Adipocyte-derived FABP4 promotes metabolism-associated steatotic liver–induced hepatocellular carcinoma by driving ITGB1-mediated β-catenin activation
Source: J Clin Invest. 2025 Dec 15;135(24):e182322. doi: 10.1172/JCI182322 (PMC12700556; doi:10.1172/JCI182322)

Unedited blots for Figure 5D

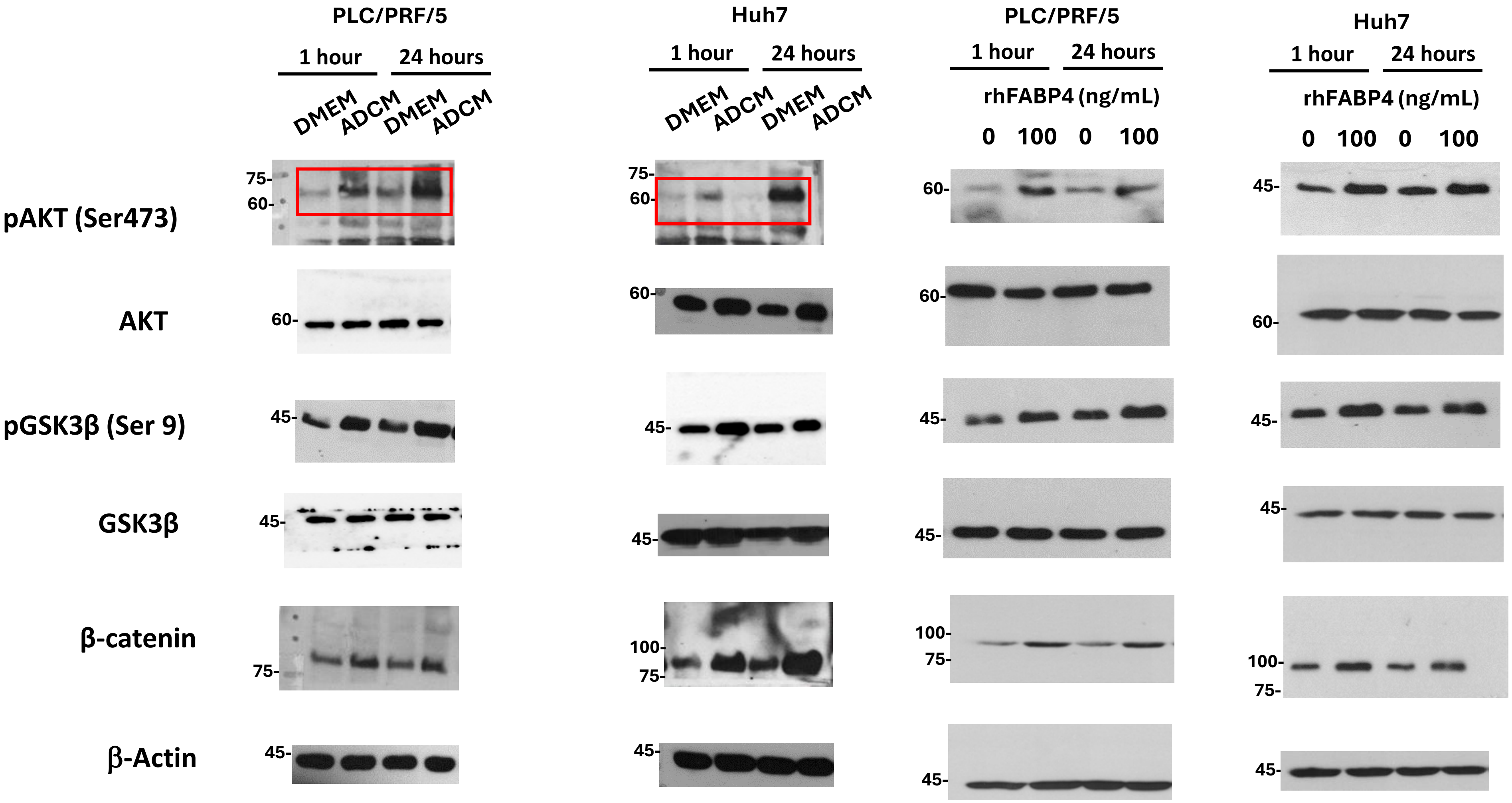

Unedited blots for Figure 6F

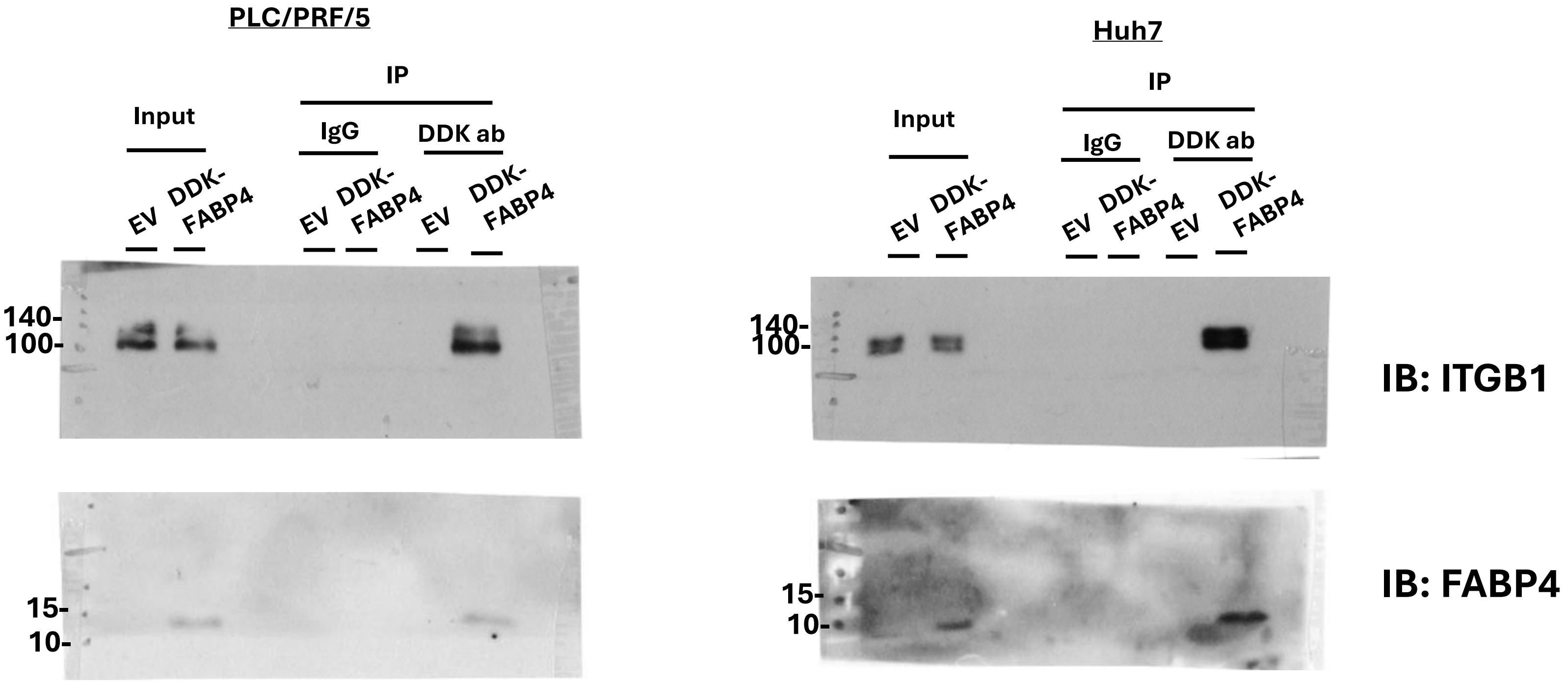

Unedited blots for Figure 6G

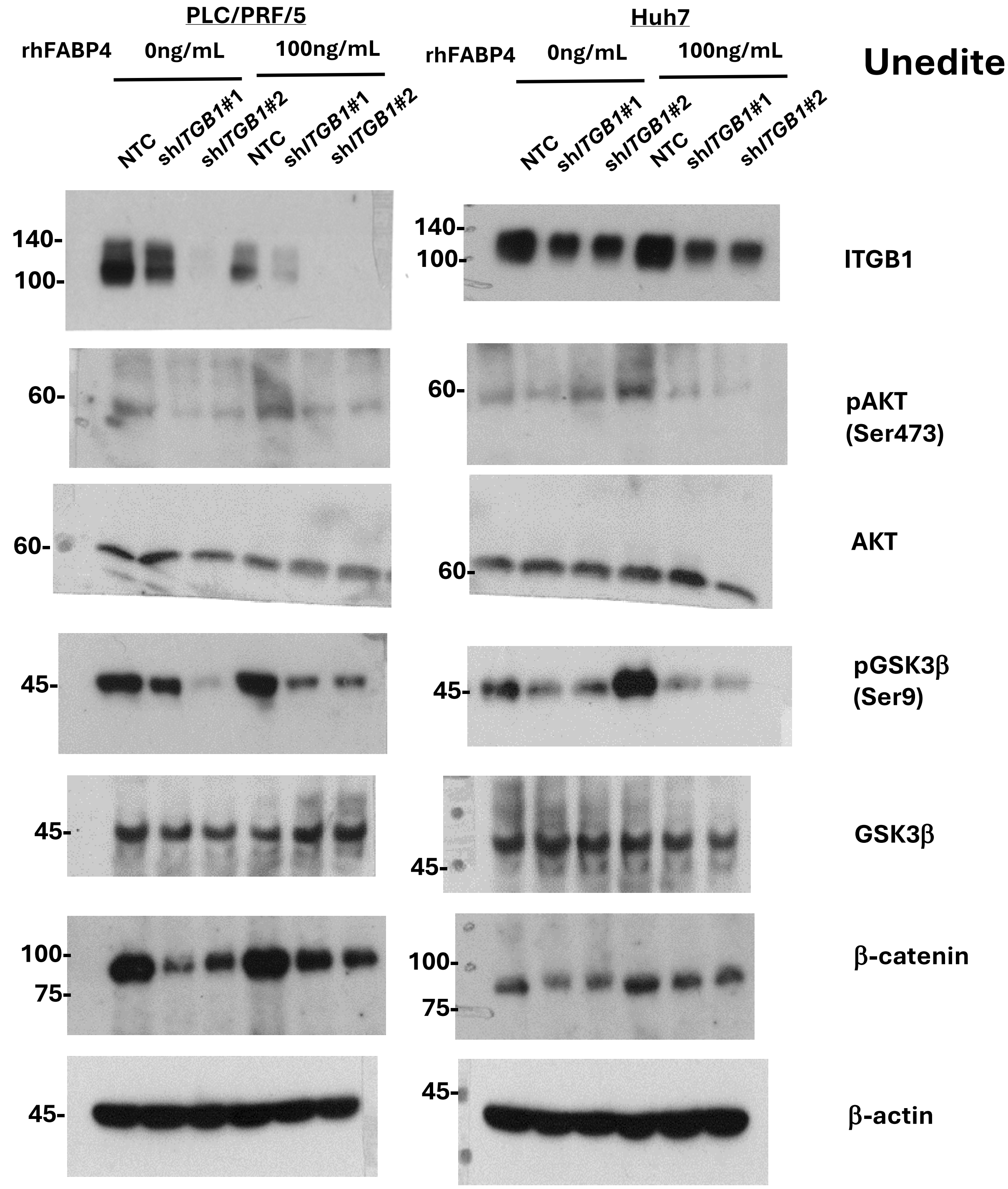

Unedited blots for Figure 7A

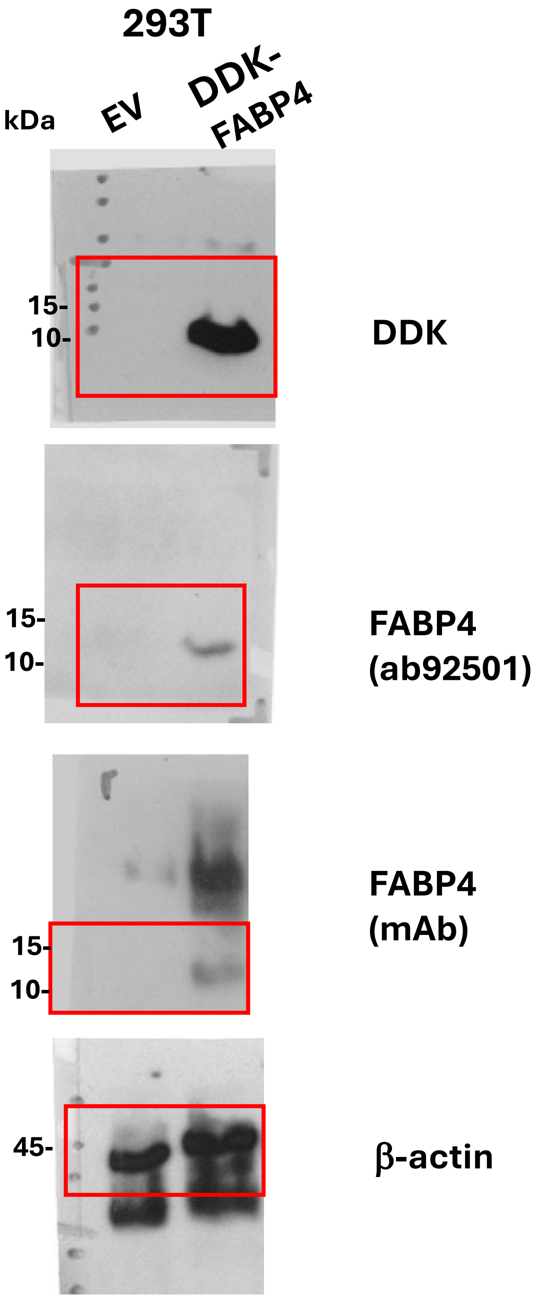

Unedited blots for Supplemental Figure 6A-C

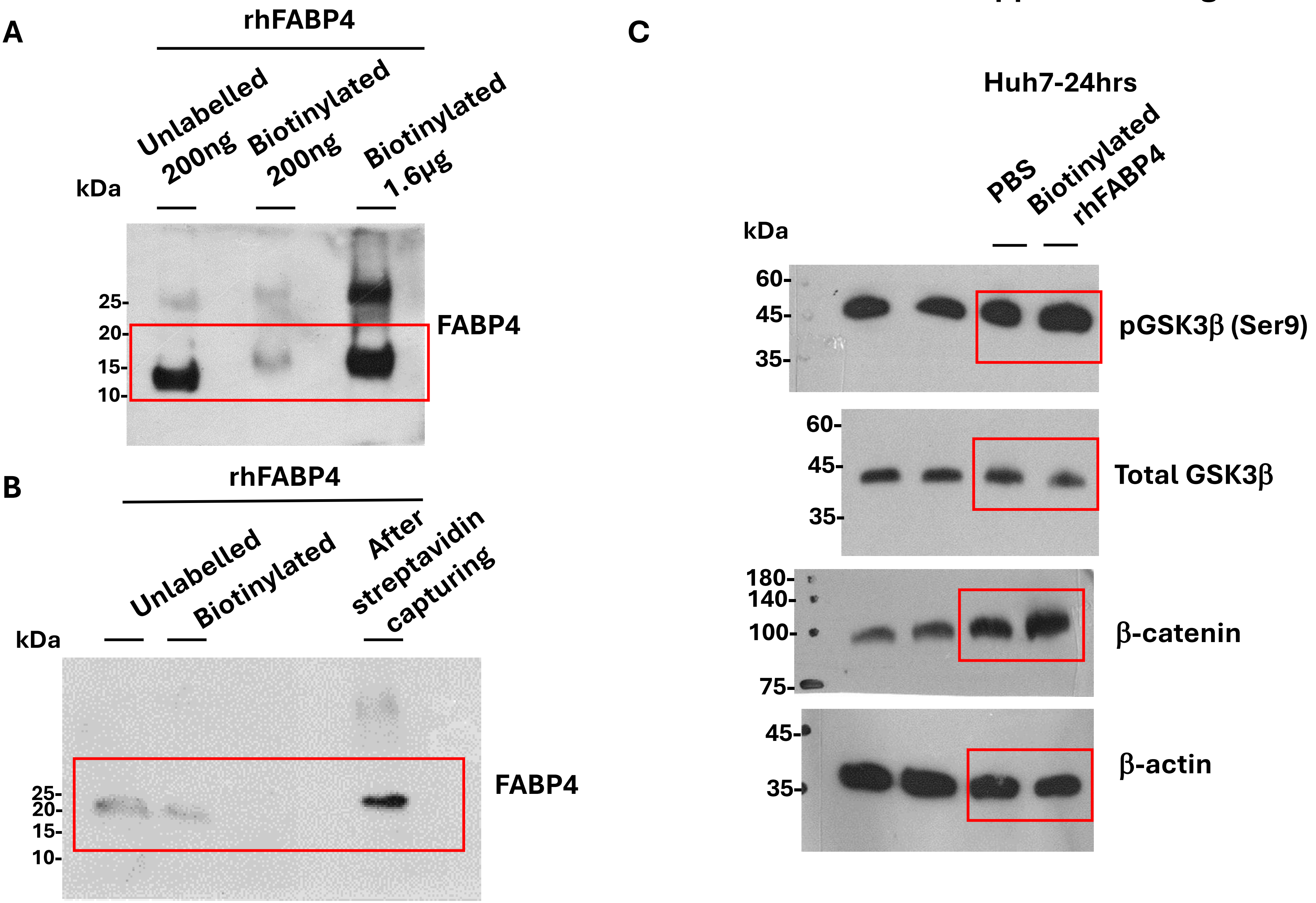

Unedited blots for Supplemental Figure 8A-B

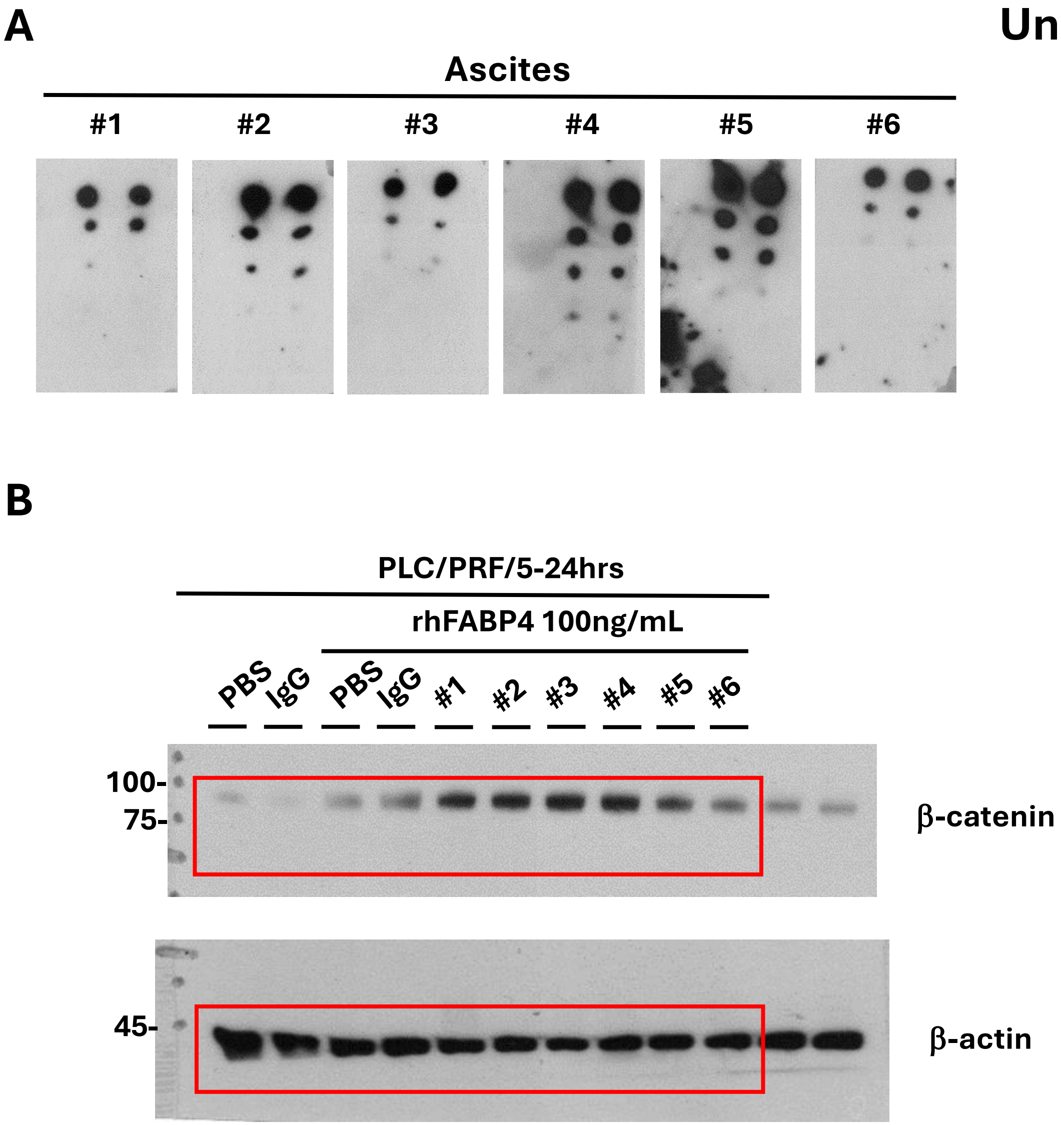

Supplement: Unedited blot and gel images [file jci-135-182322-s278.pdf]
